# Cartilaginous Extracellular Matrix Enriched with Human Gingival Mesenchymal Stem Cells Derived “Matrix Bound Extracellular Vesicles” Enabled Functional Reconstruction of Tracheal Defect

**DOI:** 10.1002/advs.202102735

**Published:** 2021-11-28

**Authors:** Tian Zeng, Pingping Yuan, Lirong Liang, Xinchi Zhang, Hui Zhang, Wei Wu

**Affiliations:** ^1^ State Key Laboratory of Military Stomatology & National Clinical Research Center for Oral Diseases & Shaanxi Key Laboratory of Stomatology Department of Anesthesiology and Department of Oral & Maxillofacial Surgery School of Stomatology the Fourth Military Medical University Xi'an 710032 P. R. China; ^2^ Department of Anesthesiology the 986th Air Force Hospital, Xijing hospital the Fourth Military Medical University Xi'an 710032 P. R. China; ^3^ State Key Laboratory of Military Stomatology & National Clinical Research Center for Oral Diseases & Shaanxi Key Laboratory of Stomatology Department of Oral & Maxillofacial Surgery School of Stomatology the Fourth Military Medical University Xi'an 710032 P. R. China; ^4^ State Key Laboratory of Military Stomatology & National Clinical Research Center for Oral Diseases & Shaanxi Key Laboratory of Stomatology Department of Anesthesiology School of Stomatology the Fourth Military Medical University Xi'an 710032 P. R. China

**Keywords:** cartilaginous extracellular matrix, ciliated columnar cells, gingiva derived stem cells, matrix bound extracellular vesicles, tracheal regeneration

## Abstract

Stem cells derived extracellular vesicles (EVs) conceive cues essential for tissue repair. Mammalian cartilaginous extracellular matrix (cECM) may not be optimally inductive for tracheal regeneration because of the granulomatous, instead of regenerative, responses in injured adult mammalian tracheas. Given the high regenerative capacity of gingiva, it is hypothesized human gingival mesenchymal stem cells derived EVs (gEVs) can induce mammalian tracheal epithelia regeneration. Coculturing chondrocytes with GMSCs produce abundant “matrix bound gEVs (gMVs)” in forming cartilaginous ECM, which are further preserved in acellular cECM (cACM) following mild, short‐period decellularization. The results show that gMVs‐cACM could be well anchored on polyglycerol sebacate microporous patch thus enforce the surgical suturability and mechanical strength. In rabbit tracheal defect, the gMVs‐cACM patch induces rapid regeneration of vascularized ciliated columnar epithelium, which supports long‐term survival of animals. gMVs‐cACM treated groups exhibit proliferation of tracheal progenitors‐basal epithelial cells, as well as, activation of JAK2/STAT1 pathway in reparative cells. This study departs from conventional focuses on tissue derived ECM and introduces a new approach for tracheal tissue regeneration.

## Graphical abstract

Human gingival mesenchymal stem cells derived matrix vesicles bound acellular cartilaginous extracellular matrix significantly promote the migration of airway basal epithelial cells and accelerate the repair of functional ciliated columnar epithelium in the process of airway regeneration. This study overcomes the long‐standing obstacles in the repair of tracheal injury, thus providing a new therapeutic conception for the segmental tracheal injury.

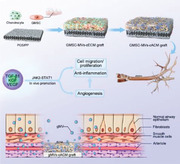

## 1 Introduction

Mammalian extracellular matrix (ECM) exhibits unique ability to promote human tissue healing following filling in defect sites or coverage on wounds.^[^ ^1^ ^]^ This regenerative potential is mainly ascribed to multiple biological factors naturally embedded in ECM, which plays a wide spectrum of modulatory roles including macrophage polarization, stem cells differentiation, cell recruitment, as well as, angiogenesis.^[^ ^2^, ^3^, ^4^ ^]^ Although detailed mechanism remains elusive, specific surface topography, mechanobiology‐related cell signaling, and tissue specific developmental cues contribute to complicated ECM‐cells interactions for driving regenerative process.^[^ ^5^ ^]^ A subgroup of extracellular vesicles (EVs), matrix‐bound vesicles (MVs) were recently identified as integral and functional component of ECM, their ability to transfer ribonucleic acid (RNA), proteins, enzymes, and lipids were believed to help regulating macrophages activation and cell differentiation induced by ECM bioscaffolds.^[^ ^6^ ^]^ Despite of this, acquiring decellularized ECM with sufficient regenerative potential remains a challenge. Bioactivity of acellular ECM (ACM) is widely influenced by donor age, tissue origin, and is always unexpectedly damaged during decellularizing process upon cycling, dramatic actions of chemical reagents and proteinase.^[^ ^7^ ^]^

Recent advances in therapeutic applications of mesenchymal stem cells (MSCs) to various diseases greatly broaden the knowledge of EVs. Compared to their parent cells, in vivo studies revealed comparable effects of stem cells released EVs on revascularization of ischemic heart,^[^ ^8^ ^]^ anti‐fibrosis in kidney,^[^ ^9^ ^]^ as well as, repair of neuron.^[^ ^10^ ^]^ Moreover, EVs may have low immunogenicity and can be safely stored without losing function.^[^ ^11^, ^12^ ^]^ The biological functions of EVs not only varied according to different cell source, but also altered with culturing environment such as hypoxia, nutrition deprivation.^[^ ^13^, ^14^, ^15^ ^]^ EVs carrying designed molecules or drugs have been incorporated into artificial grafts such as wound dressing to improve diabetic ulcer,^[^ ^16^ ^]^ and titanium implant to improve osteointegration.^[^ ^17^ ^]^ In such biomimetic systems, anti‐inflammatory and angiogenic factors embedded in nanovesicles proved their efficacy in augmenting reparative potential of scaffolds.^[^ ^18^ ^]^ Despite of the encouraging results, compromised stability and viability, as well as, low retention rate in defects remained obstacles to clinical translation.

The oral mucosa presents strong reparative ability, even under contaminated environment. MSCs derived from gingival lamina propria (GMSCs) have been proven to own strong anti‐inflammatory and regenerative potential,^[^ ^19^ ^]^ moreover, GMSCs show the stability of putative MSC markers in long‐term cultures.^[^ ^20^ ^]^ GMSCs derived EVs (gEVs) can replicate the unique biological effects of the protocell, and demonstrated their efficacy in promoting wound healing and immune regulation.^[^ ^10^, ^21^, ^22^ ^]^ We believe that the EVs from gingival stem cells are more likely to augment regenerative potential of ACM than those from fibrosis‐prone tissues. Besides, mature tissues or organs are relatively thick and thus typically require the involvement of detergents or other potent chemicals for their decellularization. This may reduce the availability and activity of trophic molecules in the ACM and alter their composition as well as 3D configuration.^[^ ^23^ ^]^ In sharp contrast, chondrocyte sheet consists of only two to three layers of chondrocytes in compact collagenous matrices and is about 100 µm thick. This enables a gentle decellularization protocol that minimizing chemical perturbations to the native ECM composition and structure.

Iatrogenic tracheal injury (ITI) is a rare but potentially life‐threatening complication of intubation, with a reported incidence of 0.05–0.37%.^[^ ^24^ ^]^ Established risk factors for ITI include inappropriate tracheal intubation, emergency or difficult airway management, and long‐term mechanical ventilation and tracheotomy in intensive care unit (ICU). Up to 12% of all positive cases for massive interstitial pneumonia, caused by the novel Coronavirus disease 2019, may need ICU admission in with possible long‐term endotracheal intubation for mechanical ventilation and subsequent tracheostomy. The most common airway‐related complications of such ICU maneuvers are laryngotracheal granulomas, stenosis, malacia, and less commonly, tracheal necrosis with tracheo‐esophageal or tracheo‐arterial fistulae.^[^ ^25^ ^]^ Surgical treatment is needed when the trachea is seriously injured, and the rapid repair of functional ciliated columnar epithelium is one of the obstacles during the repairment, which is also the key factor to restore the normal function of the airway.

Here, we established a protocol to fabricate a tracheal patch of cartilaginous ACM (cACM) bounded with GMSCs derived “matrix‐bound vesicles” (gMVs‐cACM), which was characterized, and examined for bioactivity on human bronchial basal epithelial cells (HBEs), as well as, the capability to promote rapid epithelial regeneration of gMVs‐cACM in vivo, using tracheal defect in rabbit as a model organ and cACM as a control. As a starting point to understand the working mechanism of gMVs‐cACM on mammalian tracheal epithelium regeneration, we also performed proteomic analysis and identified the role of janus kinase 2‐signal transducer and activator of transcription 1 (JAK2‐STAT1) signaling pathway in gMVs‐cACM induced regeneration of ciliated columnar epithelium. This proof‐of‐concept study explores the potential of gMVs‐cACM as a new biological material for tracheal tissue regeneration, departing from conventional focuses on decellularized tissues or organs.

## 2 Results

### 2.1 Design of a Surgically Suturable, Acellular Cartilaginous Extracellular Matrix Anchored Patch

**Figure 1:**
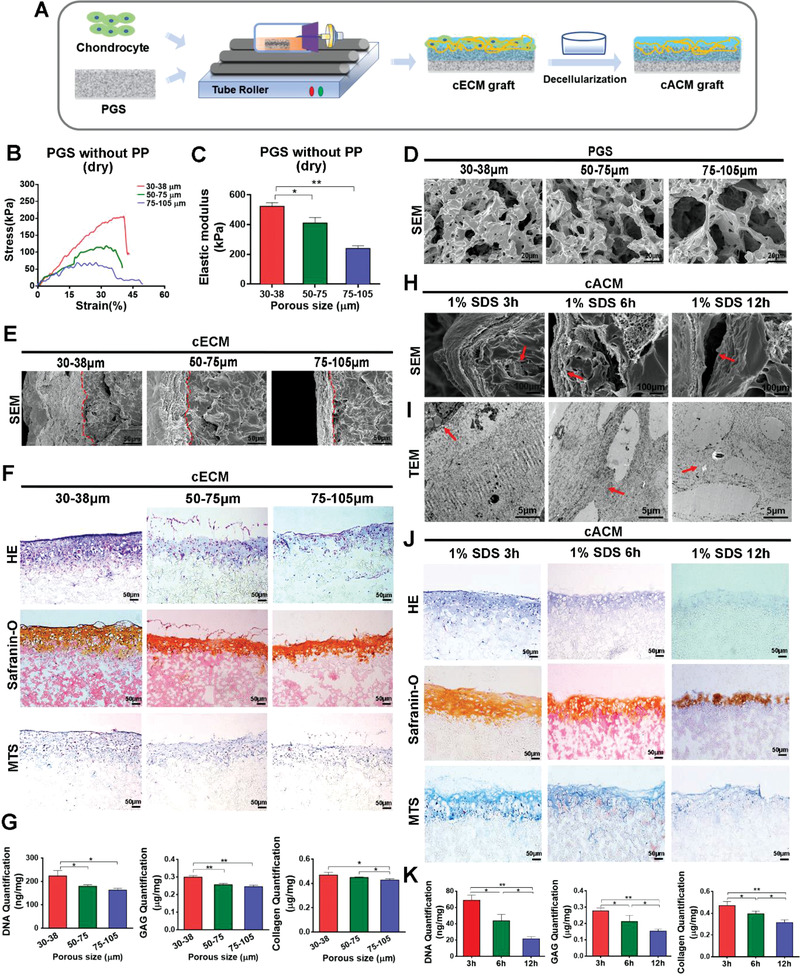
Design of a surgically suturable, acellular cECM anchoring patch by dynamic culture of chondrocytes in vitro and decellularization process. A) Schematic description of dynamic culture of chondrocytes on PGS scaffolds and fabrication of the cACM grafts. B) The stress–strain curves of 30–38, 50–75, and 75–105 µm pore size PGS scaffolds at axial direction showed that PGS with small pore size acquired the highest UTS. C) The elastic modulus of PGS were inversely proportional to the pore size (*n* = 5 independent samples, *: *p* < 0.05, **: *p* < 0.01). D) SEM images of morphology for 30–38, 50–75, and 75–105 µm pore size PGS scaffolds. E) SEM examination visualized the cECM anchoring on PGS scaffolds (Red dotted line: The junction of cECM and PGS). F) H&E, Safranin‐O, and MTS staining of cECM graft showed PGS with 30–38 µm pore size was more conductive to chondrocytes proliferation and ECM deposition than the other two scaffolds. G) Quantitative comparison of DNA, GAG, and collagen content in cECMs on different PGS scaffolds. (*n* = 5 independent samples, *: *p* < 0.05, **: *p* < 0.01). H,I) Comparing microstructures of cACMs resulted from different decellularizing processes through SEM and TEM examination. J) H&E, Safranin‐O, and MTS staining of the cACM graft showed the superior decellularizing effect in 6 h group. K) Comparison of DNA, GAG, and collagen content of the cACM graft under different decellularize procedures (*n* = 5 independent samples, *: *p* < 0.05, **: *p* < 0.01). Data were represented as the means ± SD for each group and significance was determined by one‐way ANOVA followed by Tukey's post hoc analysis.

In consistence with designing criteria, the chondrocytes were seeded on polyglycerol sebacate (PGS) porous scaffolds, and dynamically cultured in a bioreactor to acquire cartilaginous extracellular matrix (cECM) anchored patch (**Figure** **1**A). Ultimate tensile strength (UTS) and Elastic modulus were inversely proportional to the pore size of the scaffolds (Figure 1B,C; UTS: 30–38 µm, 200.9 ± 3.598 kPa, 50–75 µm, 112.4 ± 4.038 kPa, 75–105 µm, 79.4 ± 0.293 kPa; Elastic modulus: 30–38 µm, 523.3 ± 14.243 kPa, 50–75 µm, 410.8 ± 21.450 kPa, 75–105 µm, 239.2 ± 10.960 kPa). The microstructure of PGS scaffolds with three pore sizes were showed by scanning electronic microscopy (SEM) in Figure 1D. After 2 weeks’ dynamic culture, it was revealed that the pore size of PGS significantly influenced chondrocyte proliferation on scaffolds, and cECM was less detached from PGS with 30–38 µm pores as compared with other two pore‐size constructs (Figure 1E,F). Furthermore, more proliferating chondrocytes and secreted ECM were presented on 30–38 µm PGS scaffold, which was confirmed by deoxyribonucleic acid (DNA) quantification (30–38 µm, 223.43 ± 13.52 ng mg^–1^; 50–75 µm, 178.91 ± 3.95 ng mg^–1^; 75–105 µm, 162.47 ± 4.55 ng mg^–1^). Glycosaminoglycan (GAG) and collage content extracted from cECM on 30–38 µm scaffold reached the highest level among all three groups (GAG, 0.298 ± 0.005 µg mg^–1^; collagen, 0.467 ± 0.012 µg mg^–1^) (Figure 1G). Taken together, PGS constructed with 30–38 µm pore size was appropriate to cell culture and cECM anchoring, we thus used this parameter as pore‐size criteria for further experiment.

The transmission electron microscopy (TEM) examination showed that in ECM formed by chondrocytes, collagen fibrils and proteoglycan globules were arranged randomly and formed lacuna for chondrocytes (Figure S1, Supporting Information). Owing to the slimness of the cECM anchored on PGS, short‐term action with 1% sodium dodecyl sulfate (SDS) solution effectively removed the cellular components while preserved the cartilaginous matrix. It was found that the microstructure of cACM and biochemical components were dramatically altered following 3, 6, and 12 h decellularizing procedures. The results indicated that more appropriate decellularizing effect could be acquired in 6 h group, as was shown in SEM and TEM examination (Figure 1H,I). SEM examination revealed that cells remained in the ECM or the pores of PGS in 3 h group, as indicated by the red arrow. The cACM was more frequently detached from the scaffold in the 12 h group, as marked by the red arrows. In the 6 h group, the cACM without residual cells tightly anchored on PGS, as indicated by the red arrow (Figure 1H). TEM examination presented the cell lacuna was surrounding by the intact collagen fibrils in 6 h group, as marked by the red arrow. Residual cells were observed in 3 h group, and the collagen fibrils of the cACM in 12 h group were obviously sparser, indicating microstructural damage of ECM, as marked by the red arrow (Figure 1I). The cACM was structurally intact with less shrinkage in 6 h decellularizing group, less staining of nuclear chromatin manifests completely removal of cellular components (Figure 1J). In contrast, 3 h SDS action resulted in abundant residue of cellular components which was stained by haematoxylin. Safranin‐O staining of the 12 h group turned out to be the lightest among all three groups, suggesting significant loss of GAG. A similar trend was also observed in MTS staining, much collagen dissolved upon the SDS soaking in the 12 h group. Quantitative analysis of cACM showed low content of DNA in 6 h and 12 group (43.58 ± 3.58 ng mg^–1^, 21.44 ± 1.16 ng mg^–1^, respectively), and higher DNA content in 3h group (68.77 ± 2.79 ng mg^–1^) (Figure 1K). With regards to the GAG and collagen content, the lowest level (GAG, 0.153 ± 0.007 µg mg^–1^; collagen, 0.313 ± 0.018 µg mg^–1^) were both presented in the 12 h group, which suggested that severe damaging of cECM following decellularization. The content of GAG and collagen in the 3h group and the 6h group were displayed in Figure 1K, respectively (3 h, 0.277 ± 0.008 µg mg^–1^, 0.469 ± 0.017 µg mg^–1^; 6 h, 0.211 ± 0.017 µg mg^–1^, 0.395 ± 0.005 µg mg^–1^). Taken together, 6 h SDS action was appropriate for decellularization of PGS anchored cECM.

### 2.2 Rational Decellularization Preserved Microvesicles Bounded with Cartilaginous Acellular Extracellular Matrix

**Figure 2:**
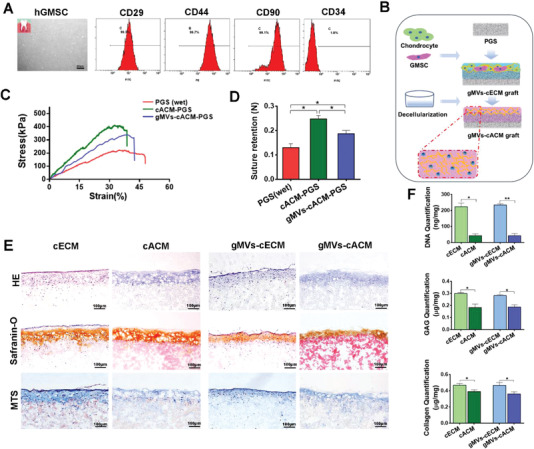
Characteristics of ECM graft formed by chondrocytes cocultured with Hu‐GMSCs. A) Flow cytometry analysis revealed Hu‐GMSCs matched the characteristics of MSCs that CD29, CD44, and CD90 were highly expressed and few cells were positive in CD34. B) Schematic illustration of dynamic coculture process for chondrocytes and Hu‐GMSCs with a ratio of 4:1 on PGS scaffold and fabrication of gMVs‐cACM graft. C) Representative stress–strain curves of PGS (wet), cACM, and gMVs‐cACM grafts showed the mechanical strength of the grafts were improved once ACM was formed, while coculturing GMSCs slightly compromised the strength of formed cACM. D) The comparison of the suture retention of PGS (wet), cACM, and gMVs‐cACM grafts (*n* = 5 independent samples, *: *p* < 0.05). E) H&E, Safranin‐O, and MTS staining of cECM grafts and gMVs‐cECM grafts which visualized the changing of collagen and GAG content after decellularization. F) The quantification of DNA, GAG, and collagen of grafts before and after the optimized decellularization procedure (*n* = 5 independent samples, *: *p* < 0.05, **: *p* < 0.01). Data were represented as the means ± SD for each group and significance was determined by one‐way ANOVA followed by Tukey's post hoc analysis.

Human gingival mesenchymal stem cells (Hu‐GMSCs) were isolated from the normal gingival tissues obtained from 10 healthy donors aged 18–25 years. Optical microscope image presented the typical spindle shape of isolated GMSCs (**Figure** **2**A). Flow cytometry analysis presented that 98.2% of GMSCs were negative for CD34, a hematopoietic cell surface marker, but consistently expressed cluster of differentiation 29 (CD29) (99.3%), CD44 (99.7%), and CD90 (99.1%) at passages 3–4. The results indicated that GMSCs could be steadily expanded in vitro and maintain their phenotypes. Furthermore, tripotential to differentiate into osteogenic cells, adipogenic cells, and chondrogenic cells suggested the stemness of cultured GMSCs (Figure S2, Supporting Information). Owing to the poor ECM producing ability, especially when cells were passaged to 3–4 generations, we failed to acquire GMSCs derived ECM after decellularization (Figure S3, Supporting Information). GMSCs‐ECM group were therefore excluded in further studies. The seeding ratio of chondrocytes and GMSCs was selected according to preliminary experimental results. In our preliminary studies, increasing the number of GMSCs significantly compromised the strength and quality of cartilaginous ECM, which led to the poor integrity of gMVs‐cACM after decellularization (Figure S4, Supporting Information). Due to the contaminated environment of airway, it is difficult for such grafts to resist infection. When the proportion of GMSCs was rationalized to 20%, the histological results showed sufficient cartilaginous ECM formed and was well preserved after decellularization. Therefore, chondrocytes and GMSCs were seeded on PGS scaffolds (30–38 µm pore size) at ratio of 4:1, 2 weeks’ dynamic culture enabled production of significant amount of cECM (Figure 2B).

After decellularization following above optimized procedure, anchoring cACM on PGS not only changed the appearance of the scaffold, but also strengthened the mechanical properties. Compared with the cACM, the ACM formed by chondrocytes cocultured with GMSCs was slightly softer, resulting the reduction of the mechanical strength of the gMVs‐cACM graft. UTS and suture retention of ACM‐grafts were enhanced as compared with PGS graft (UTS: PGS, 216.0 ± 9.263 kPa, cACM‐PGS, 410.4 ± 7.143 kPa, gMVs‐ cACM‐PGS, 342.3 ± 11.4 kPa; suture retention: PGS, 0.13 ± 0.007 N, cACM‐PGS, 0.25 ± 0.006 N, gMVs‐cACM‐PGS, 0.19 ± 0.006 N), which made them favorable for surgical handling and resistance to tracheal pressure (Figure 2C,D). Histological examination revealed decellularization with 1% SDS for 6 h removed cellular components of both groups effectively (Figure 2E). According to the results of SEM and histological staining in Figures 1H,J and 2E, the thickness of cACM was about 100 µm and that of gMVs‐cACM was about 80 µm. DNA content of cACM grafts and gMVs‐cACM grafts decreased significantly from 223.43 ± 13.52 ng mg^–1^ and 234.36 ± 4.49 ng mg^–1^ to 42.86 ± 6.48 ng mg^–1^ and 42.59 ± 7.80 ng mg^–1^ (Figure 2F), respectively. Decellularization significantly compromised GAG and collagen content in the cACM and gMVs‐cACM grafts. The GAG content of cACM and gMVs‐cACM grafts decreased from 0.299 ± 0.005 µg mg^–1^ and 0.282 ± 0.005 µg mg^–1^ before decellularization to 0.183 ± 0.017 µg mg^–1^ and 0.187 ± 0.011 µg mg^–1^ after decellularization. The collagen content of the cACM and gMVs‐cACM grafts decreased from 0.468 ± 0.013 µg mg^–1^ and 0.466 ± 0.020 µg mg^–1^ to 0.391 ± 0.012 µg mg^–1^ and 0.360 ± 0.016 µg mg^–1^, respectively. Interestingly, for GAG and collagen contents, no significantly differences were detected between cACM and gMVs‐cACM grafts (*p* > 0.05).

**Figure 3:**
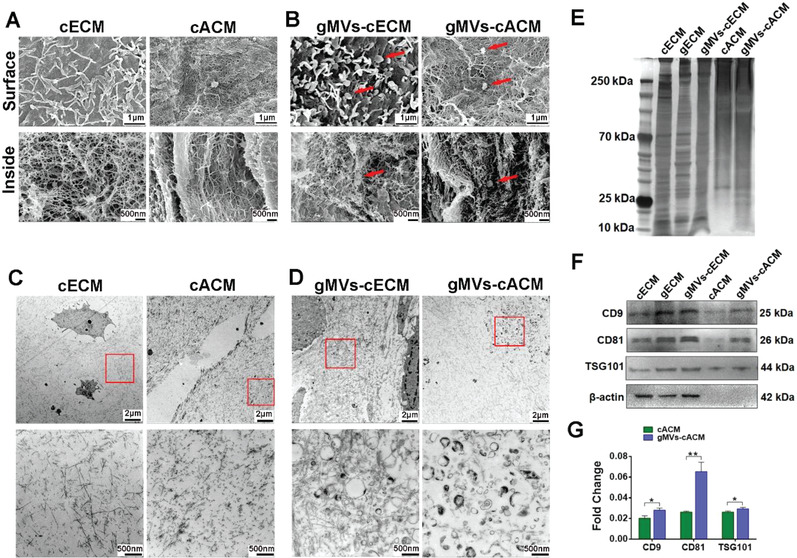
GMSCs‐MVs were wrapped in the nano‐fibers of cECM. A) SEM examination showed the superficial and interior microstructure of cECM and cACM were mainly composed of nanofibrous structure. B) SEM examination showed that many vesicles were bounded onto the fibers on the surface and inside of the gMVs‐cECM, as well as, gMVs‐cACM, as indicated by the red arrows. C) TEM examination presented nanofibrous microstructure of cACM turned to be slightly sparser and looser than that of cECM. D) TEM examination showed a large number of gMVs were wrapped by collagen fibers in the gMVs‐cECM and gMVs‐cACM. E) The silver staining showed the differences of protein expression in ECM from different groups before and after decellularization. F,G) Western blotting revealed the expression of the EVs marker proteins, CD9, CD81, and TSG101 in the gMVs‐cECM before and after decellularization. The expression level of these markers was analyzed semi quantitatively (*n* = 3 independent samples, *: *p* < 0.05, **: *p* < 0.01). Data were represented as the means ± SD for each group and significance was determined by Student's *t*‐test analysis between cACM group and gMVs‐cACM group.

Ultrastructural examination through SEM and TEM revealed the contribution of GMSCs to cECM constituents. The microstructures of cECM and cACM were mainly composed of collagen fibrils on the surface and inside, while vesicles were rarely observed (**Figure** **3**A). Coculturing GMSCs with chondrocytes produced more micro or nano‐vesicles, which were shown to be bound among collagen fibrils, as indicated by the red arrows (Figure 3B). TEM examination also showed that collagen fibers were the main component of cECM and cACM (Figure 3C). Decellularization decreased gMVs in the cACM at least to some extent. However, clusters of gMVs could be frequently detected in cACM cocultured with GMSCs, ranging in sizes from 50 to 500 nm, which seemed to be wrapped and fixed by the fibrous structure of the cACM, as displayed by the high magnification of the red box (Figure 3D). Moreover, these vesicular structures usually existed at a distance of 20–30 micrometers away from the cell lacuna (Figure S5A, Supporting Information). Furthermore, these gMVs were also found in acellular matrices infiltrating in PGS pores (Figure S5B, Supporting Information).

In order to explore whether MVs can affect the proteomic composition of cACM, silver staining was performed to visualize contained proteins between cACM and gMVs‐cACM (Figure 3E). The results suggested that the proteins from cACM and gMVs‐cACM were markedly different. The diversity also existed between the cECM and gMVs‐cECM. The difference may be caused by GMSCs since the protein expression of pure GMSCs‐ECM (gECM) was quite different from that of cECM. Furthermore, master markers of EVs, CD9, CD81, and tumer susceptibility gene 101 (TSG101) were identified through Western blotting (Figure 3F,G). There was significant difference in the semi‐quantitative analysis of the three marker proteins between cACM and gMVs‐cACM groups. The contrast of these markers between cECM and gMVs‐cECM, as well as, cACM and gMVs‐cACM, revealed that GMSCs contribute significant amount of gMVs in cACM, which could be well preserved through “cell sheet decellularization” procedure. gMVs were isolated following enzymatic digestion and gradient ultracentrifugation, then characterized by nanoparticle tracking analysis (NTA), TEM examination and western blot for protein markers. NTA showed the size and the intensity distribution of gMVs (Figure S6A, Supporting Information), indicating that the size of gMVs mostly ranged from 50 and 300 nm. The morphology of gMVs examined with TEM showed the typical bilayer‐membrane vesicles (Figure S6B, Supporting Information). Western blotting analysis showed that gMVs expressed the specific markers of EVs (CD9, CD81, and TSG101) (Figure S6C, Supporting Information).

### 2.3 Proteomics Analysis of the gMVs‐cACM Graft Proved Its Pro‐Migration and Proliferative Potential

**Figure 4:**
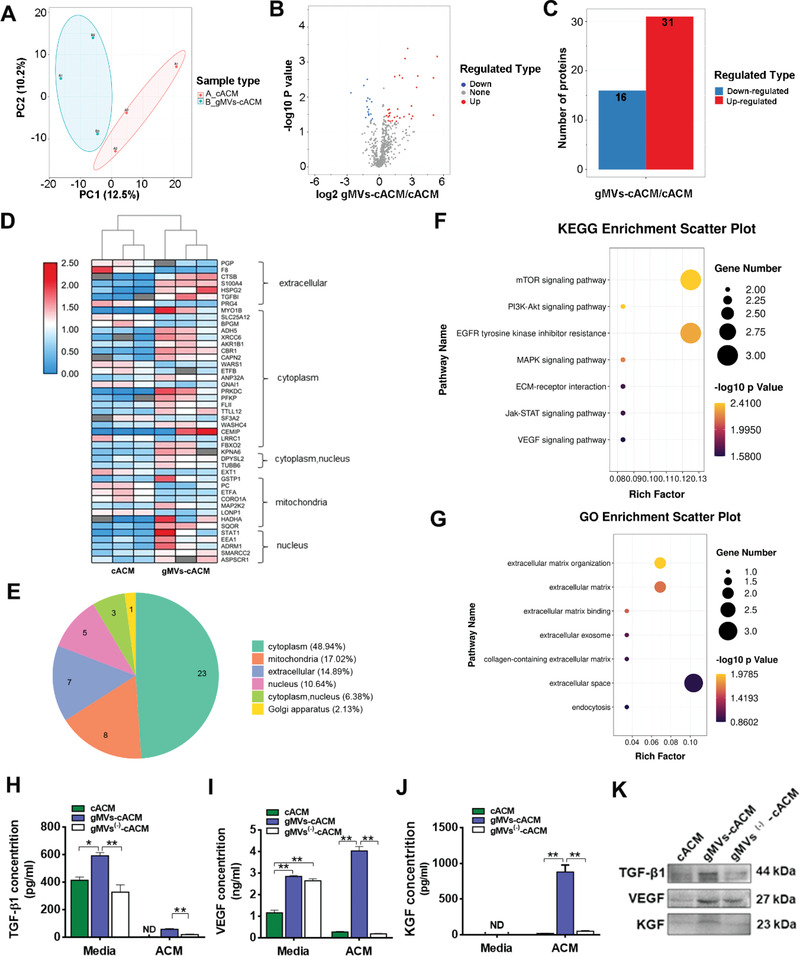
The proteomics dataset analyzed for biological significance of cACM graft and GMSCs derived MVs enriched cACM graft. A) The principal component analysis indicated the high aggregation degree of samples in each group (*n* = 3 in each group). B,C) Volcano plot showed differentially expressed proteins between cACM and gMVs‐cACM (Red dots: Up regulation; Blue dots: Down regulation), which were quantified with histogram. D) Heatmap represented the cluster analysis of the differentially expressed proteins in cACM grafts and gMVs‐cACM grafts. E) Prediction and classification of subcellular localization showed the differentially expressed proteins mainly concentrated in the cytoplasm and extracellular. F) The enrichment analysis of KEGG pathway suggested the differentially expressed proteins were annotated in the cell proliferation and angiogenesis, such as, JAK‐STAT signaling pathway and VEGF signaling pathway. G) The GO annotation of differentially expressed proteins showed their different biological roles in the ECM. H–J) The contents of TGF‐*β*1, VEGF, and KGF in gMVs‐cACM grafts measured by ELISA were significantly higher than those in cACM grafts, which could be declined by the EVs secreting inhibitor GW4869 in gMVs ^(‐)^‐cACM group. (ND: Non‐detectable; *n* = 3 independent samples, *: *p* < 0.05, **: *p* < 0.01). K) The expression of TGF‐*β*1, VEGF, and KGF were examined by Western blotting. For (F,G), Fisher's exact test was used to test the differentially expressed proteins. The corrected *p* < 0.05 was considered significant and expressed as −log10 (*p*‐value). The size of the circle represented the degree of enrichment. For (H–J), data were represented as the means ± SD for each group and significance was determined by one‐way ANOVA followed by Tukey's post hoc analysis.

After thorough quality control assessment of 6 samples from gMVs‐cACM grafts and cACM grafts (*n* = 3 in each group), the comprehensive proteomic profile of grafts was investigated using 4D label free LC‐MS/MS based proteomics analysis. 753 of 1559 commonly identified proteins were quantifiable, which was selected for differential expression analysis. Principal component analysis (PCA), which was conducted to reflect the range of variation among samples within the group, revealed distinct distribution of proteins between the two groups (**Figure** **4**A). There were 47 differential expression proteins identified between cACM and gMVs‐cACM grafts, as shown in the quantitative volcano map (Figure 4B). The red dots showed 31 proteins which was up‐regulated, and the blue dots showed 16 proteins which was down‐regulated (Figure 4C). The heat map of cluster analysis showed the enrichment of differential proteins in subcellular localization of cells (Figure 4D), which suggested that differential proteins were strongly, positively correlated with “extracellular constituent” and “cytoplasm,” rather than “nuclear composition” related. In the extracellular domain, several proteins were closely related to cell differentiation and proliferation between two groups, such as transforming growth factor β1 (TGF‐*β*1), S100 calcium binding protein A4 (S100A4). The pie chart showed the proportions of differential proteins in cytoplasm and extracellular were 48.94% and 14.89%, respectively (Figure 4E), indicating the differential proteins were probably related to EVs. All the identified and differentially expressed proteins were annotated by the kyoto encyclopedia of genes and genomes (KEGG) and gene ontology (GO) enrichment analysis. KEGG analysis showed that some signaling pathways related to cell proliferation (mitogen‐activated protein kinase, MAPK, JAK‐STAT), differentiation (the phosphatidylinositol 3‐kinase /Akt,PI3K‐Akt), and angiogenesis (vascular endothelial growth factors (VEGF)) were significantly different, indicating that gMVs‐cACM was likely to exert physiological activity in these aspects (Figure 4F). GO enrichment analysis showed differentially expressed proteins were related to extracellular structures and functions (Figure 4G). Since the ECM is the basic microenvironment for cell migration and proliferation, it can be inferred that the two kinds of ACM grafts may exert different biological activities. In addition, cytokines closely related to angiogenesis, cell differentiation, and proliferation in the process of tissue regeneration, were detected by enzyme‐linked immuno sorbent assay (ELISA) to verify the results of proteomics (Figure 4H–J). Compared with cACM graft, the concentrations of TGF‐*β1*, VEGF, and keratinocyte growth factor (KGF) in gMVs‐cACM graft were significantly higher, which decreased sharply in the gMVs ^(‐)^‐cACM group by administrating EVs secreting inhibitor GW4869 during the culture period. This finding proved that the bioactive components were chiefly brought by GMSCs‐MVs in cACM. The concentrations of VEGF and TGF‐*β*1 in the conditional medium (CM) of gMVs‐cACM group were higher than that of cACM group, while KGF was nondetectable (ND) in the CM. The expression of TGF‐*β*1, VEGF, and KGF were also verified by Western bolting (Figure 4K), which showed the same trend with ELISA.

### 2.4 gMVs‐cACM Graft Exhibits Bioactivity In Vitro

As the first step to investigate the regenerative potential of gMVs‐cACM for mammalian trachea, we examined their bioactivity on the proliferation and migration of HBEs in vitro. As examined by Cell Counting Kit‐8 (CCK‐8) assay, the viability of HBEs seeded on gMVs‐cACM patch was significantly higher than that of cACM group from the 3rd day to the 7th day after seeding (Figure S7A,B, Supporting Information). Furthermore, cytokeratin 5+8 (CK5+8) immunostaining was performed to verify the proliferation of HBEs on grafts (Figure S7C,D, Supporting Information). Notably, significantly more CK5+8 positive cells were found on the gMVs‐cACM grafts than cACM grafts and PGS grafts, which confirmed the promoting role of gMVs in cell proliferation. The same trend was also identified by the DNA quantification (Figure S7E, Supporting Information).

**Figure 5:**
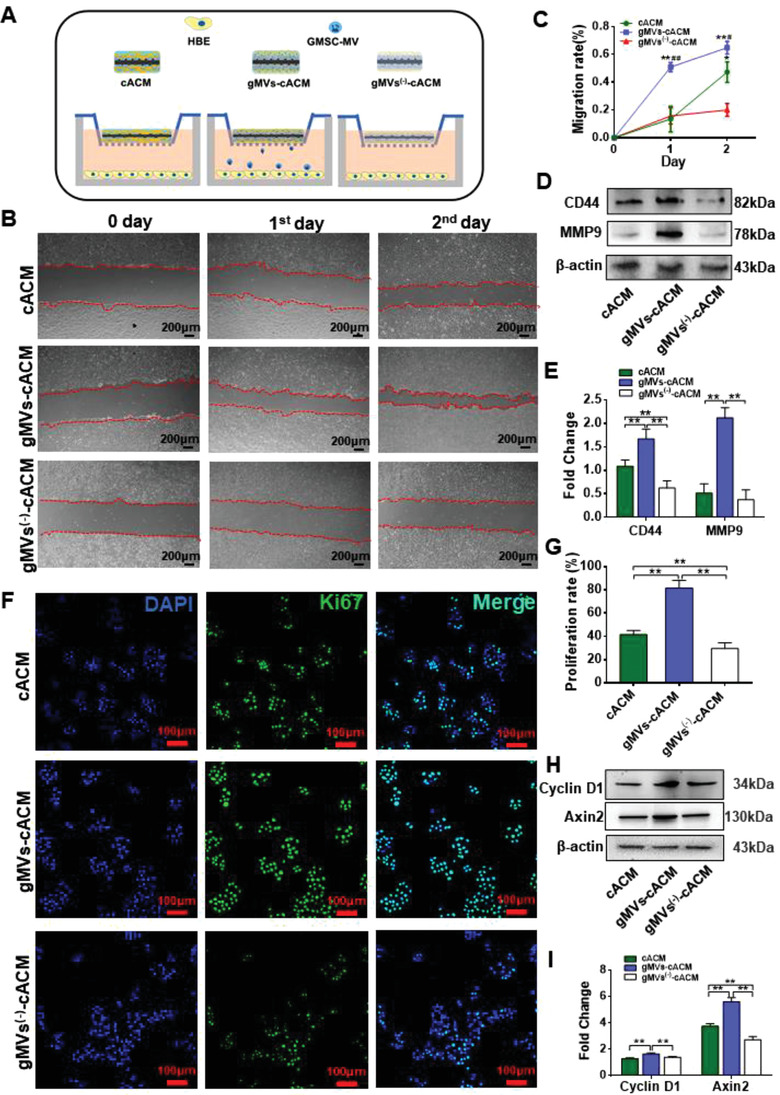
The promotion of gMVs‐cACM graft on migration and proliferation of HBEs in vitro. A) Schematic illustration of the coculturing HBEs with cACM graft, gMVs‐cACM graft, and gMVs^(‐)^‐cACM graft by Transwell plates. B) The cell scratch assay revealed the promoting effect of gMVs‐cACM graft on the migration of cocultured HBEs, which was significantly inhibited once EVs secretion was inhibited by GW4869 in gMVs^(‐)^‐cACM group. C) Comparison of the migration rates of HBEs in different groups at the 1 d and 2 d in the cell scratch assay (*n* = 6 independent samples, *: versus gMVs^(‐)^‐cACM group, *: *p* < 0.05, **: *p* < 0.01; #: versus cACM group, #: *p* < 0.05, ##: *p* < 0.01). D,E) Western blotting showed the different expression of cell migration related proteins CD44 and MMP9 in HBEs cocultured with cACM graft, gMVs‐cACM graft, and gMVs^(‐)^‐cACM graft, suggesting that the expression of migration related proteins was reduced by the inhibition of EVs secretion. (*n* = 3 independent samples, *: *p* < 0.05). F) Ki67 immunofluorescence staining revealed the increased proliferation of HBEs in gMVs‐cACM group at 48 h, which was decreased in gMVs^(‐)^‐cACM group. G) The proliferation rate of HBEs in gMVs‐cACM group was highest among the three groups based on positive cell number of Ki67 after 48 h of coculture. (*n* = 6 independent samples, **: *p* < 0.01). H,I) Western blotting showed the high expression of cell proliferation related proteins Cyclin D1 and Axin2 in HBEs cocultured with the gMVs‐cACM graft (*n* = 3 independent samples, **: *p* < 0.01). Data were represented as the means ± SD for each group and significance was determined by one‐way ANOVA followed by Tukey's post hoc analysis.

The cell scratch assay was used to simulate the crawling process of epithelial cells adjacent to grafts margins to study the effects of gMVs (**Figure** **5**A). The gMVs‐cACM graft significantly accelerated the migration of HBEs and started to repair the scratch at the 1st day, but this trend was impeded once the release of EVs was pharmacologically inhibited by GW4869 (Figure 5B,C). The migration ability of HBEs in gMVs^(‐)^‐cACM group was even lower than that in cACM group. It was speculated that a few EVs released by chondrocytes was inhibited at the same time, which reduced the ability of cACM to induce cell migration to a certain degree. CD44 is a transmembrane glycoprotein that is commonly expressed on the surface of both immune and epithelial cells of airway, and plays vital role in cell proliferation, migration, cell‐ECM adhesion. It is reported that increased CD44 expression in bronchial epithelium suggested the enhanced reparative activity.^[^ ^26^, ^27^ ^]^ Owing to intrinsic bioactivity hold in cACM in promoting cell proliferation and migration, the CD44 expression in HBEs of cACM group was also maintained at a certain level, although significantly lower than that in gMVs‐cACM group (Figure 5D). However, once the gMVs release was inhibited by GW4869 in grafts, the promoting role on HBEs, manifested by CD44 expression, reduced significantly. Matrix metalloproteinase 9 (MMP‐9), a member of the MMP family, is involved in the breakdown of extracellular matrix in many physiological conditions such as embryonic development, reproduction, and tissue healing.^[^ ^28^ ^]^ WB analysis of pro‐migration related proteins showed that MMP‐9 of HBEs were highly expressed in gMVs‐cACM group as compared with cACM group (Figure 5D,E). Inhibiting secretion of EVs sharply decreased the expression of CD44 and MMP9 of HBEs. Ki67, a classical cell proliferating related antigen,^[^ ^29^ ^]^ was stained to reveal that proliferation rate of HBEs in gMVs‐cACM group was significantly higher than that in cACM group, while it declined significantly once EVs release was inhibited (Figure 5F,G). WB confirmed the up‐regulation of Cyclin D1 and Axin2, two proliferation related protein, in HBEs cocultured with gMVs‐cACM graft (Figure 5H,I). gMVs are actually a subset of GMSCs derived EVs (gEVs), which has shown its reparative potential in wound healing.^[^ ^30^ ^]^ As a positive control, we also examined the biological effects of gEVs on HBEs in vitro. It was found that the migration and proliferation of HBEs increased significantly, once the gEVs contained medium was fed (Figure S8, Supporting Information), which confirmed the unique effects of GMSCs derived EVs on HBEs. Taken together, the promoting effect of proliferative and migration could be augmented by cACM bound gMVs.

### 2.5 Toxicity Evaluating of gMVs‐cACM Graft In Vivo

The toxicity of gMVs‐cACM graft was evaluated in vivo by the histological analysis. It is known that PGS has been widely studied in vascular regeneration and bone repair, and has demonstrated its biocompatibility.^[^ ^31^ ^]^ The acellular matrix derived from organisms were already developed for clinical application. Here, the superiority over previous procedure was using milder detergent to reduce toxic effects during decellularization. In addition, decellularizing procedure may significantly reduce the immune rejection, tumorigenicity caused by the cellular composites. As expected, all the mice survived without immunological rejection, the wounds healed normally without infection. Histologically, no apparent morphological changes were detected in the harvested myocardium, liver, spleen, lung, renal, and brain through 1 and 3 weeks (Figure S9A, Supporting Information). The mild inflammation was identified around grafts at 1 week while diminished at 3 weeks, neovascularization and cell infiltration in scaffolds were observed in gMVs‐cACM grafts since the first week (Figure S9B, Supporting Information, as shown by the black stars). No apparent pathological changes such as tissue necrosis, granulomas formation around grafts were identified, which indicated that the implantation of the gMVs‐cACM grafts did not result in systemic and local toxicity. Moreover, at 3 weeks, the grafts were almost degraded and replaced by newly formed vascularized fibrous tissue, indicating that the grafts were highly biocompatible and bioactive.

### 2.6 Release of Vesicles from gMVs‐cACM Graft In Vivo and In Vitro

In transwell system (Figure S10A, Supporting Information), the DiO labeled gMVs were engulfed in cocultured HBEs after 24 h's coculturing, and accumulated over time, suggesting gMVs could be released from the prepared grafts and play roles through endocytosis (Figure S10B, Supporting Information). In contrast, HBEs cocultured with cACM and PGS/polypropylene (PP) grafts were negative in these vesicles. The quantification of the DiO labeled gMVs endocytosed by HBEs in vitro was showed in Figure S10C, Supporting Information. Furthermore, in vivo monitoring of gMVs release was performed by the bioluminescence imaging (*n* = 3 independent samples, Figure S10D, Supporting Information). In vivo, although fluorescence signals of DiO presented attenuating trend at the implanting region through 1 d, 3 d, and 7 d in the gMVs‐cACM groups (Figure S10E, Supporting Information), the controlled release of gMVs from the grafts was confirmed. As controls, no obvious signals were detected in implanting regions in cACM and PGS groups.

### 2.7 Repairing Tracheal Defect with gMVs‐cACM Graft Improves Functional Recovery of Airway

**Figure 6:**
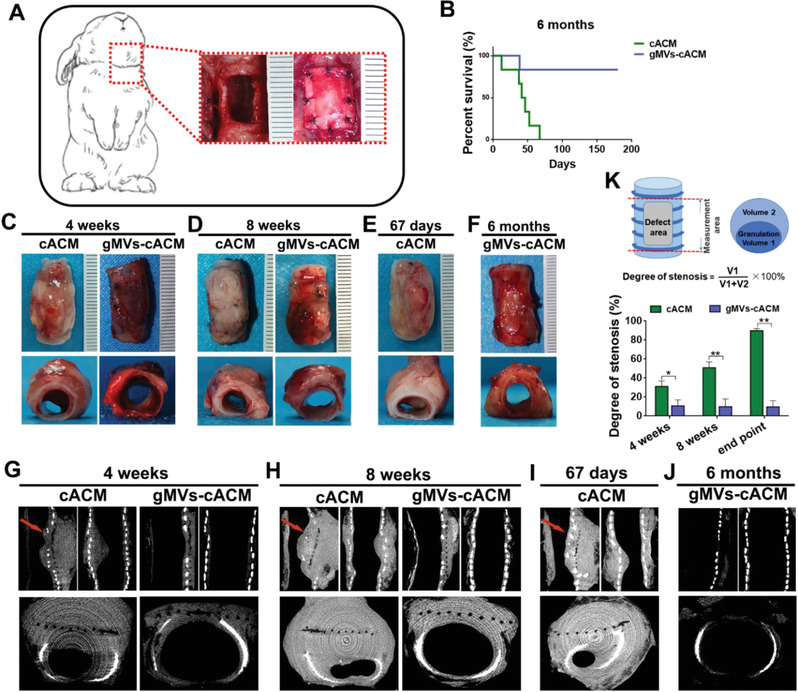
In vivo transplantation of ACM grafts in the tracheal defect model. A) Schematic description of the ACM graft implantation for the tracheal anterior wall defect model (10 × 5 mm^2^). B) The survival rate of animals was 83.33% in gMVs‐cACM group at 6 months after transplantation whereas no animals survived more than 67 days in cACM group (*n* = 6 independent samples). C–F) Gross appearances of reconstructed tracheas in cACM group and gMVs‐cACM group at 4 weeks, 8 weeks, and endpoint after transplantation. G–J) CT images showed the reconstructed tracheal lumen and red arrows indicated the sites of stenosis in cACM group. K) Schematic diagram showed the degree of airway stenosis was calculated by 3D reconstruction image of CT scanning. The degree of tracheal stenosis in cACM group were significantly higher than that in gMVs‐cACM group at 4 weeks, 8 weeks, and endpoint (*n* = 3 independent samples, *: *p* < 0.05, **: *p* <0.01). Data were represented as the means ± SD and significance was determined by Student's *t*‐test analysis. The survival rates were analyzed by Log‐rank test.

The efficacy of gMVs‐cACM on tracheal repair was investigated in adult rabbit “anterolateral tracheal wall” defect model (**Figure** **6**A). For the functional studies, the rabbits in the 6 months group implanted with gMVs‐cACM grafts lived through 6‐month experimental duration. In contrast, the survival period of animals implanted with PGS was 7–12 days, and cACM group can live no more than 67 days (Figure 6B). The autopsy revealed that the main lethal factor in PGS group was tracheal obstruction and asphyxia caused by increased sputum after infection of the airway (Figure S11A, Supporting Information). Most animals in cACM group died at 6–7 weeks postoperatively, and suffered fatal respiratory distress caused by chronic tracheal stenosis. The harvested samples were first subjected to gross examination, including superficial appearance, luminal patency and diameter. It's found that mild stenosis with no sputum retention was observed in the luminal surface of the cACM graft at 4 weeks after implantation. In the gMVs‐cACM group, the luminal surface was very clear (Figure 6C). At 8 weeks, the lumen in cACM group was significantly narrowed, and is almost blocked by growing granulation (Figure 6D). In contrast, the glossy luminal surface was presented in gMVs‐cACM group, and the grafts integrated with the adjacent trachea without disruption or granulation formation (Figure 6C,D,F). Microcomputed tomography (CT) examination quantitatively characterized stenosis following different grafts implantation (Figure 6G–J). Since the 4th week to ending point, the degree of stenosis in cACM group were 31.20%, 50.89%, and 89.97% at 4 weeks, 8 weeks, and 67 days, respectively, suggesting that the inflammatory granulation tissue continued to proliferate in the airway after operation, leading to progressive stenosis in the airway lumen, which eventually led to the death of animals. Conversely, the degree of stenosis in gMVs‐cACM group were 10.65%, 9.97%, and 9.61% at 4 weeks, 8 weeks, and 6 months, respectively, indicating that the process of airway repair was fast and stable, with few adverse postoperative complications (Figure 6K).

### 2.8 gMVs‐cACM Graft Accelerates Regeneration of Respiratory Epithelium in Tracheal Defect

**Figure 7:**
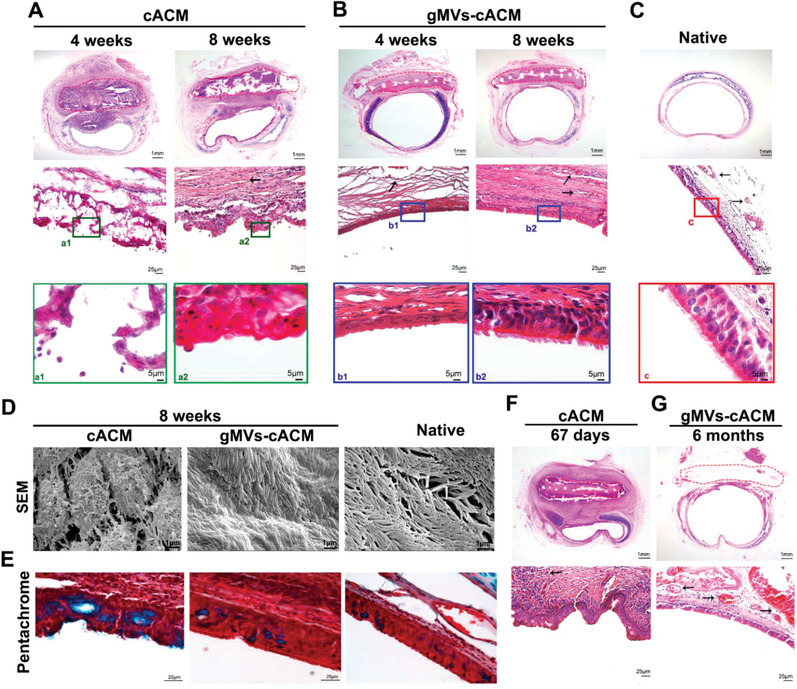
gMVs‐cACM graft accelerated the regeneration of respiratory epithelium on tracheal defect. A) H&E staining of the reconstructed trachea in cACM group at 4 weeks and 8 weeks after transplantation showed the disturbance of airway epithelial regeneration in the luminal side of the graft that circled by the red dotted line. B) H&E staining showed the morphology and arrangement of the regenerated epithelium in gMVs‐cACM group were consistent with that of autologous epithelium at 4 weeks and 8 weeks postoperatively. C) H&E staining of the normal native trachea provided a reference to identify the regenerated epithelium in each group. D) SEM examination showed luminal appearance of the reconstructed trachea at 8 weeks postoperatively. E) The modified Pentachrome staining manifested the density of goblet cells (blue) in the regenerated epithelium of cACM group and gMVs‐cACM group at 8 weeks after transplantation. F,G) H&E staining of the regenerated epithelium were quite different in morphology in cACM group at 67 days and in gMVs‐cACM group at 6 months. The black arrows indicated the neo‐vessels formed in the mucosa of the luminal side of the graft.

Hematoxylin and Eosin (H&E) staining on serially sectioned trachea revealed that gMVs‐cACM treatment altered pathological remodeling of tracheal defect. PGS grafts failed to regenerate any tissues owing to acute infection and sputum blocking (Figure S11C,D, Supporting Information). Through 4 to 8 weeks after surgery, defects treated with cACM graft presented overgrowth of granulation tissue around the grafts (**Figure** **7**A). In contrast, a layer of polarized cells extended from the surrounding native epithelium and covered gMVs‐cACM grafts since the 4th week, which further presented cilium structures at 8 weeks, just similar to native trachea (Figure 7B,C). These findings were further confirmed by luminal examination by SEM (Figure 7D). The density, morphology and length of cilium appeared similar with those of the native trachea. Pseudostratified airway epithelium with mucus‐containing goblet cells and ciliated airway epithelial cells were observed throughout the native trachea lumen by the Movat‐Russell modified Pentachrome stain (Figure 7E). In cACM group, goblet cells stained as blue color were more frequently observed than ciliated columnar epithelium; in gMVs‐cACM group, ciliated columnar epithelium was closely arranged and goblet cells were evenly distributed among them. Newly formed epithelium failed to show polarity which was characterized by ciliated columnal epithelia even at 67 days postoperatively due to the persistent inflammation caused severe stenosis in trachea of cACM group (Figure 7F; Figure S12A,B, Supporting Information). In contrast, 6‐month samples of gMVs‐cACM group showed that PGS had been completely degraded, and the airway epithelial structure remained intact. The grafts underwent consistent host remodeling including cell infiltration and vascularization, without any inflammatory granulation nodule through the 6 months (Figure 7G, Figure S12A,B, Supporting Information).

**Figure 8:**
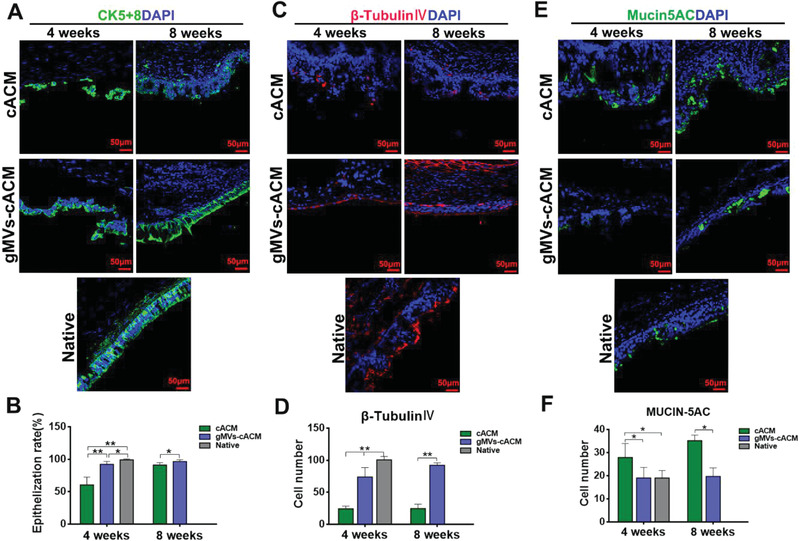
Immunofluorescence staining of the functional epithelium in reconstructed trachea. A,B) Immunofluorescence staining of reconstructed trachea revealed the regeneration of CK5+8 positive epithelial basal cells (green), and the epithelialized rate was calculated according to the CK5+8 positive cell at 4 weeks and 8 weeks after transplantation in cACM group and gMVs‐cACM group (*n* = 6 independent samples, *: *p* < 0.05, **: *p* < 0.01). C,D) Immunofluorescence staining of *β*‐Tubulin‐IV positive ciliated columnar epithelium (red) and the quantification of the positive cell number in gMVs‐cACM group were significantly higher than those of cACM group at 4 weeks and 8 weeks after transplantation (*n* = 6 independent samples, **: *p* < 0.01). E,F) Immunofluorescence staining of Mucin5AC positive goblet cells (green) in the regenerated epithelium and the quantification of them showed the increased expression in cACM group at 4 weeks and 8 weeks after transplantation (*n* = 6 independent samples, *: *p* < 0.05). Data were represented as the mean ± SD for each group, and significance was determined by one‐way ANOVA followed by Tukey's post hoc analysis or Student's *t*‐test analysis.

Immunofluorescence staining was performed to clarify the cell phenotypes in repairing epithelium (**Figure** **8**). The subtypes of cytokeratin, CK5+8 is the main skeleton protein in keratinocytes, which has been identified as marker of basal epithelial cells. The main function of this structural protein, which is closely related to the proliferation and differentiation of epithelial cells, is to maintain the integrity and continuity of epithelial tissue.^[^ ^32^ ^]^ As shown in Figure 8A, at 4 weeks postoperatively, both cACM group and gMVs‐cACM group presented in‐growth and migration of CK5+8 positive cells in the defecting area. Obviously, gMVs‐cACM grafts induced more complete epithelial coverage, as revealed by more continuously green stained line. These cells continue to increase through 8 weeks and became more aligned. In contrast, CK5+8 positive cells covering cACM grafts were interrupted with unstained cells, proliferation was slow through 8 weeks, with poor alignment and uneven epithelial thickness. The rate of epithelialization, which was calculated by the ratio of the length of the positive cell line to the length of the defect in the section, was significantly different at 4 weeks between two groups (Figure 8B, cACM: 60.38 ± 4.923%, gMVs‐cACM: 92.13 ± 1.788%). *β*‐Tubulin‐IV was used to display the regenerated ciliated columnar epithelium.^[^ ^33^ ^]^ gMVs‐cACM grafts significantly induced more ciliated columnar epithelium differentiation than cACM grafts, (Figure 8C,D, 4 weeks: cACM, 24.33 ± 1.68/field, gMVs‐cACM, 78.33 ± 5.99/field; 8 weeks: cACM, 24.50 ± 2.79/field, gMVs‐cACM, 92.00 ± 1.59/field, native:100.50 ± 2.17/field). Goblet cells, secreted mucin to wrap foreign bodies, were marked by Mucin‐5AC. It is found that more goblet cells presented in neo‐epithelium on cACM graft than that in gMVs‐cACM group, which was probably due to inflammation stimulus (Figure 8E,F, 4weeks: cACM, 27.83 ± 2.48/field, gMVs‐cACM, 19.00 ± 1.88/field; 8 weeks: cACM, 35.17 ± 0.98/field, gMVs‐cACM, 19.67 ± 1.52/field, native: 19.01 ± 1.342/field). No positive cells of these three markers were detected in PGS group, suggesting the difficulty to achieve airway epithelial repairment by orthotopic transplantation of bare materials (Figure S11E, Supporting Information).

### 2.9 JAK2‐STAT1 Pathway Play Roles in gMVs‐cACM Mediated Tracheal Repair

**Figure 9:**
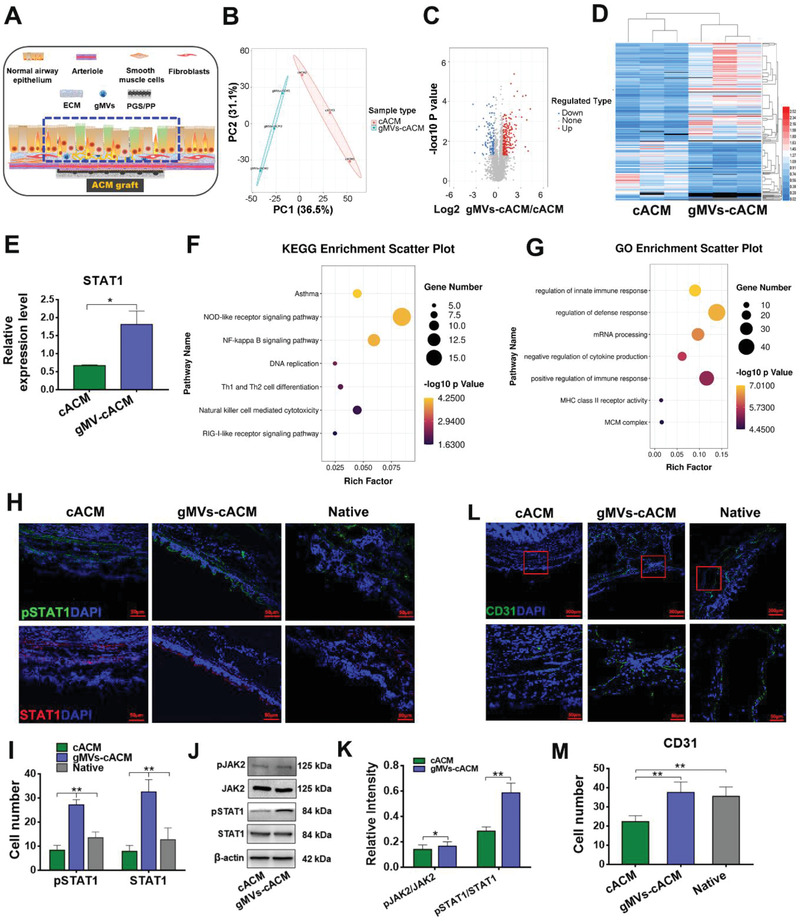
JAK2‐STAT1 signaling pathway participated in gMVs‐cACM mediated tracheal repair. A) The sampling site of the regenerated epithelial layer covering the graft for proteomics analysis was circled by the blue dotted line. B) The PCA plot indicated the high aggregation degree of proteins in cACM group and gMVs‐cACM group. C) The volcano plot showed the differentially expressed proteins in cACM group and gMVs‐cACM group (Red dots: Up regulation; Blue dots: Down regulation). D) Heatmap depicted a significant change of proteins in neo‐epithelial tissue between cACM and gMVs‐cACM group. E) The relative expression levels of STAT1 protein in cACM and gMVs‐cACM group were significantly different according to the proteomics analysis (*n* = 3 independent samples, *: *p* < 0.05). F) The KEGG analysis showed the differentially expressed proteins mainly enriched in the signaling pathway related to the inflammation and cell proliferation, such as, NOD‐like receptor signaling pathway, NF‐kappa B signaling pathway, and DNA replication. G) The GO annotation analysis indicated the differentially expressed proteins were involved in the regulation of immune response and defense response. H,I) Immunofluorescence staining of pSTAT1 (green) and STAT1 (red) showed the different expression in the neo‐epithelium at 3 weeks after the transplantation in cACM and gMVs‐cACM group, and the quantification of positive cells showed the up‐regulated expression in gMVs‐cACM group (*n* = 6 independent samples, **: *p* < 0.01). J,K) Western blotting verified the activation of JAK2‐STAT1 signaling pathway in gMVs‐cACM group at 3 weeks after the transplantation (*n* = 3 independent samples, *: *p* < 0.05, **: *p* < 0.01). L,M) Immunofluorescence staining of CD31 (green) characterized the vascularized level of the neo‐mucosa in gMVs‐cACM group was significantly higher than that of cACM group at 3 weeks after the transplantation (*n* = 6 independent samples, **: *p* < 0.01). Data were represented as the mean ± SD for each group and significance was determined by one‐way ANOVA followed by Tukey's post hoc analysis or Student's *t*‐test analysis.

The epithelium samples of the reconstructed trachea at 3 weeks postoperatively were assigned for 4D label free quantitative proteomics analysis (*n* = 3 in each group, **Figure** **9**A). A total of 1015 proteins were identified, of which 696 contained quantitative information. The repeatability of protein quantification was evaluated by PCA (Figure 9B). The volcano map showed that there were a considerable number of differential expression proteins between gMVs‐cACM group and cACM group (Figure 9C). Heatmap exhibited proteins having a significant change in the neo‐epithelial layer between gMVs‐cACM group and cACM group at early remodeling stage (Figure 9D). STAT1 protein was detected to be highly expressed in gMVs‐cACM group according to the cluster analysis (Figure 9E). Since JAK2‐STAT1 pathway plays an important role in cell proliferation and differentiation, it may participate in the regeneration of airway epithelium. The pathway enriched by KEGG analysis was highly correlated with immune response and cell proliferation, such as, NF‐kappa B and DNA replication signaling pathway (Figure 9F). Meanwhile, GO analysis also enriched a large number of differential proteins in biological processes related to self‐defense response and immune response (Figure 9G). These findings suggested that gMVs‐cACM graft may regulate the immune response and inflammatory response, thus rationalize microenvironment for tissue repair. Immunofluorescence staining of pSTAT1 and STAT1 verified the different expression of the proteins in the neo‐epithelium on the different grafts (Figure 9H,I). Western blotting confirmed the involvement of JAK2‐STAT1 signaling pathway to promote epithelial regeneration in gMVs‐cACM group (Figure 9J,K). Immunofluorescence staining of CD31 showed vascularization of the neo‐mucosal layer in gMVs‐cACM group, which suggested timely blood perfusion could be recovered since the 3rd weeks following gMVs‐cACM treatment (Figure 9L,M; cACM: 22.33 ± 1.25/field, gMVs‐cACM: 37.50 ± 1.21/field, Native: 35.50 ± 1.99/field). The results of proteomics and ELISA for ACM grafts confirmed high expression level of VEGF in gMVs‐cACM, which is a favorable agent to promote angiogenesis after transplantation. Furthermore, by pretreating HBEs with AG490 in vitro, the inhibitor of JAK2, the migration and proliferation of HBEs were declined compared with gMVs‐cACM group, which further confirmed the participation of this signaling pathway in the repair process of epithelium (Figure S13A–D, Supporting Information).

**Figure 10:**
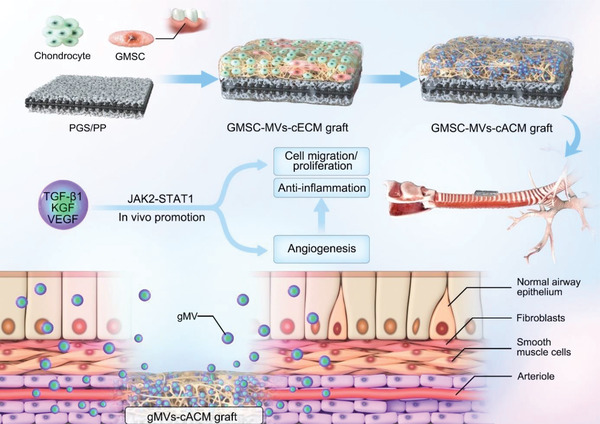
Schematic diagram of the underlying mechanism by which the gMVs‐cACM graft promoted the rapid regeneration of ciliated columnar epithelium in tracheal defect model in vivo.

Since the previous studies have reported that TGF‐*β*1 could crosstalk with JAK2‐STAT1 signal pathway in cells,^[^ ^34^ ^]^ to examine if JAK2‐STAT1 signal pathway activation during epithelial cell regeneration was regulated by TGF‐*β*1, we assessed the migration and proliferation of HBEs cocultured with the gMVs‐cACM graft with intervention of TGF‐*β*1 neutralizing antibody. The in vitro experimental design and mechanism were shown in Figure S14A, Supporting Information. HBEs cocultured with gMVs‐cACM graft exhibited enhanced migrating ability as compared with that of the gMVs‐cACM + anti‐TGF‐*β*1 group (Figure S14B,C, Supporting Information). The cell viability and proliferation of HBEs increased significantly in gMVs‐cACM group (Figure S14D,E,F, Supporting Information). However, these effects were impeded after administrating the neutralizing antibody of TGF‐*β*1. To further verify whether JAK2‐STAT1 signal pathway, which participated in the elevated migration and proliferation of HBEs, was manipulated by TGF‐*β*1 released from the gMVs, the protein levels of JAK2 and STAT1 were examined by Western blot. The JAK2‐STAT1 signal pathway was up‐regulated in HBEs under the actions of gMVs‐cACM. Conversely, the neutralizing antibody of TGF‐*β*1 weakened the activation of JAK2‐STAT1 signal pathway (Figure S14G,H, Supporting Information). These results proved that gMVs‐cACM grafts may promote the proliferation and migration of HBEs through TGF‐*β*1 mediated JAK2‐STAT1 signal pathway activation.

## 3 Discussion

Although human or mammalian tracheal epithelial cells proliferate and self‐renew through adulthood, regenerating respiratory epithelium remains challenging once full‐thickness, critical sized damage in tracheal wall occurred.^[^ ^35^ ^]^ Although decellularized allogenous trachea, polymeric prosthesis or tissue engineered grafts presented advantages in recovering mechanical properties and structural integrity in tracheal reconstruction, insufficient re‐epithelialization, or slow mucosalization of tracheal substitutes is always life‐threatening owing to bacteria invasion, granulation related stenosis, excessive scarring, and mucus impaction.^[^ ^36^, ^37^, ^38^ ^]^ Therefore, regenerating respiratory epithelium on grafts lumen must be completed as quickly as possible. Combining bioactive ACM and mechanically tough polymer is believed to be a promising strategy to reconstruct tracheal defects.^[^ ^39^ ^]^

As compared with collagenous ECM from fibroblasts or smooth muscle cells, cartilaginous ECM presents much stiffness and elasticity, thus can firmly attach the porous patch.^[^ ^37^, ^40^ ^]^ cECM has demonstrated potential to recruit the host's endogenous cells in joint repair, this approach did not require additional cell transplantation thus holds great clinical potential.^[^ ^41^ ^]^ Previously, we found that the chondrocyte sheets could be sufficiently decellularized by using low‐concentration (1%) SDS thus maintained the integrity and bioactivity of cECM. In vivo study also proved that decellularized chondrocyte sheet was superior in preserving bioactive molecules of cECM, and led to sufficient repair of articular cartilage.^[^ ^42^ ^]^ However, the mechanical strength of artificial ACM is relatively weak, which cannot provide acquired support and suturing strength for trachea reconstruction. Therefore, PGS/PP scaffold was utilized as matrices carrier and enabling ACM to play its biological effect. By this study, we surprisingly found “cell sheet decellularizing” protocol well preserved stem cells MVs in ACM. To the best of our knowledge, this study is the first to demonstrate that GMSCs derived EVs could be naturally bounded with cECM through simple “cell sheet” coculturing procedure. Coculturing GMSCs and chondrocytes in aggregation enabled self‐assembly of MVs in the forming ECM, which is a new approach to acquiring stem cells derived EVs. As compared with stem cells from other sources, gingiva is abundant, readily accessible and easily obtainable through minimally invasive cell isolation techniques.^[^ ^43^ ^]^ Transcriptomic analysis has revealed that GMSCs secreted EVs containing several growth factors such as TGF‐*β*1, fibroblast growth factor, and VEGF implicated in angiogenetic process.^[^ ^44^ ^]^ In this study, we further investigated proteomic profiles of gMVs‐cECM after decellularization. We found that the composition of gMVs‐cACM was significantly different from cACM, with more anti‐inflammation and pro‐proliferation proteins, which enhanced the regeneration potential of cACM. As expected, gMVs‐cACM presented stronger pro‐proliferative and chemotactic effects on HBEs as compared with cACM, whereas pharmacologically inhibiting EVs production significantly compromised this efficacy. The differences in cell proliferation and migrating rate suggested differential rescue and/or inductive capacities with each “matrix bound EVs” treatment under deprived growth conditions. Complex components were included in ECM, such as MVs, cytokines, peptides, as well as, microRNAs. The proteomic analysis for constructed grafts in this study revealed distinct protein profiles between the cACM and gMVs‐cACM groups. Owing to cytokines enriched in ECM, cACM itself hold potential to promote tissue repair. However, our in vivo and in vitro experiments, both presented superiorities of gMVs‐cACM grafts over cACM grafts in promoting HBEs proliferation and tracheal repair. Therefore, it was convinced that gMVs or cytokines from GMSCs played vital roles. KEGG analysis revealed signaling pathways relating to cell proliferation (MAPK, JAK‐STAT), differentiation (PI3K‐Akt) and angiogenesis (VEGF) were significantly different among groups, indicating that gMVs may exert physiological activity through these pathways. Moreover, along with significantly compromised effects of gMVs‐cACM after administrating GW4869, inhibiting MVs release from cocultured cells also reduced the level of TGF‐*β*1, VEGF and KGF in gMVs‐cACM grafts, therefore inversely verified the unique actions of gMVs. Collectively, it was believed that the angiogenic factors and epithelium related growth factors contained in gMVs play vital roles in promoting regeneration of ciliated epithelium as well vascularized trachea wall, which should be further identified and verified in mechanism.

Surgically suturing ACM‐PGS patches on anterolateral tracheal defects showed that incorporating GMSCs derived MVs in cACM, enabled endogenous regeneration of ciliated respiratory epithelium. We observed notably increased proliferation and migration of HBEs, a residential epithelium progenitor, in gMVs‐cACM group. CK5+8, a master marker of epithelial cell, persistently expressed in both seeded and proliferated HBEs on gMVs‐cACM in vitro. Proteomic analysis for gMVs‐cACM revealed existence of multiple proteins involving in the proliferation related signaling pathway, including TGF‐*β*1, S100A4, PI3K‐Akt pathway, MAPK pathway, et al., which may play roles through paracrine. Histological tracing of tracheal healing manifested vascularized, functional re‐epithelialization was derived from neighboring tracheal wall. It is known that airway basal epithelial cell could differentiate into goblet cell or ciliated columnal epithelium,^[^ ^45^ ^]^ which functionalized as secreting and removing mucous respectively. Since the 4^th^ week, migrating cells on gMVs‐cACM not only expressed CK5+8 but also re‐expressed *β*‐tubulin IV, master marker of ciliated columnal cells. These cellular regenerative responses following gMVs‐cACM grafting contributed to the overall structural regeneration and functional recovery, which correlated with ≈92.13% recovery of defective regions. Under identical conditions, cACM yielded only 60.38% functional recovery with limited improvement of animal survival and minimal differentiation of ciliated epithelium, consistent with literature reports on mammalian ECM in repairing tracheal defect.^[^ ^46^ ^]^ Our data indicated that incorporating GMSCs derived MVs into cACM sufficiently augmented differentiating potential toward ciliated columnal cells and angiogenesis beneath the epithelial tissue. Together, these results suggested the superior efficacy of gMVs‐cACM in the surgical reconstruction of tracheal defects.

gMVs‐cACM likely exerted its activities via multiple mechanisms in the re‐epithelialization, for example, the alteration of immunological reactions in host body has been manifested by proteomic analysis, which was also widely confirmed in other tissue regeneration.^[^ ^21^, ^47^ ^]^ On the other hand, we detected the upregulation of JAK2‐STAT1 in healing epithelium on gMVs‐cACM graft, one of a handful of conserved signal transduction pathways required for normal development and adult physiology, as well as for regenerative responses during infection and injury.^[^ ^48^ ^]^ In this study, we confirmed its positive roles in the proliferation and migration of HBEs. Blocking JAK2‐STAT1 signal transduction, through administering AG490, a small‐molecule tyrphostin that selectively inhibits STAT autophosphorylation, significantly inhibit proliferation and migration of HBEs. These results indicated the participation of JAK2/STAT1 signaling pathway in gMVs‐cACM bioactivity on respiratory epithelium repair. Using rabbit model, we focus more on rational design of gMVs‐cACM grafts, as well as, verifying its promoting roles in tracheal regeneration. Although representative mechanism involving TGF‐*β*1 and JAK2‐STAT1 was proved by proteomic analysis and in vitro TGF‐*β*1 blocking test, it was acknowledged that gene knockout mice would be required to verify the mechanism in further studies. Moreover, the specific drugs targeting JAK2‐STAT1 pathway should also be developed and tested to enhance reparative potential of trachea in vivo.

A unique challenge to tracheal graft is its needs to contact air once implanting in airway. Bacterial colonization and infection are the main causes for grafts failure. In our study, foreseeable infection occurred in PGS group and resulted animal death in 12 days. These results showed poor reparative potential of PGS graft alone, as demonstrated by frequently occurred infection as well as high mortality of animals. cACM itself was very weak in mechanical strength, and using cACM without PGS/PP support failed to meet the surgical requirements of tracheal transplantation. Biomimicking native tracheal structure, combining microporous lumen and macro‐porous sublayer is believed to reduce luminal contamination and promote vascularization. With the aid of polymeric or metal mesh, collagen foam infused with fibrin gel, or non‐woven mesh mediated cell produced ECM have demonstrated promising results in vivo.^[^ ^39^, ^49^, ^50^ ^]^ Departing from above designs, we fabricated cACM‐PGS construct. Owing to the similar elasticity and adequate stiffness, the firm anchorage between cACM and PGS pores was well maintained, which was critical to physical integrity and biological efficacy. Although incorporating cACM on PGS membrane significantly enhanced animal survival, cACM alone was not sufficient to prevent bacterial invasion and grafts infection, resulting in only 50% survival rate at 8 weeks. In sharp contrast, gMVs‐cACM thoroughly prevented infection related granulation. We believe at least three factors may contribute to the success of gMVs‐cACM: 1) Accelerated basal epithelial cells migration, which has been demonstrated in the migration assay in vitro; 2) Enhanced differentiation of ciliated columnal epithelium, which lead to the functional recovery of bacterial removal; 3) Promoted angiogenesis under the epithelium, which may enforce the immunologic defense ability of defective regions. Although whether gMVs‐cACM is effective in segmental tracheal defect or not remains to be further investigated, this result is the first step to show the simple incorporation of GMSCs derived MVs into cACM could augment anti‐infectious ability of decellularized grafts. Epithelialization of tracheal graft has been found in various decellularized biological grafts, and the thick ECM layer may act as physical shield to prevent bacterial invasion.^[^ ^39^, ^51^ ^]^ In contrast, ECM in cACM graft is very thin, even superficial degradation of matrices may expose the PGS materials and cause bacterial invasion. Severe tissue swelling around cACM patch was observed through 4 weeks to endpoint, the necropsy revealed the formed abscess around implant due to the wound exposure to luminal cavity. Therefore, faster epithelial ingrowth is dramatically required in ECM anchoring PGS patches, which also highlights the significance GMSCs‐MVs in ECM mediated fast epithelialization.

The results of this study demonstrated that the artificial scaffold, when modified with cartilaginous ECM enriched with GMSCs derived MVs, is able to be completely incorporated by the host trachea and ciliated epithelia regenerated rapidly. Although the defect size in this study was not very large, the prospect of clinical application in ITI is considerable. Meanwhile it provides a promising step toward the potential final goal of tracheal reconstruction with scaffold in the case of a long segmental defect. Considering that most of the graft failure in long tracheal segment reconstruction is due to the lack of mucosal regeneration, we believe our study represents an advancement in the development of synthetic material grafting without cell seeding. Further study with large animal subjects is needed to verify the enhancement of mucosal regeneration in segmental tracheal reconstruction.

## 4 Conclusion

We identified the gMVs could be retained in the cACM by the mild artificial decellularization. The gMVs‐cACM graft created a brand new and highly effective interface, which could regulate the inflammation responses and promote the angiogenesis for rapidly regeneration of ciliated columnar epithelium thus offering a rational and promising strategy for trachea replacement (**Figure** **10**).

## 5 Experimental Section

### Fabrication of Polyglycerol Sebacate/Polypropylene Scaffold

PGS prepolymer was synthesized as previously described,^[^ ^52^ ^]^ then was dissolved in tetrahydrofuran of 10% w v^−1^. The scaffold was produced by dropping PGS solution into a fused salt template casted from a 1.5 × 1.5 × 0.05 cm^3^ cuboid mold, with PP mesh stent sandwiched between 2 layers salt template for animal surgery. The prepolymer scaffold was crosslinked by heated at 150 °C under vacuum for 24 h, then salt was removed by dissolving the scaffold in deionized water. The different pore size of PGS scaffold was determined by the crystal sizes of sodium chloride particles which were 30–38, 50–75, and 75–100 µm.

### Mechanical Tests

Since the ultimate tensile force of PP mesh is beyond the measuring range of the instrument, the mechanical properties of pure PGS were tested by uniaxial tensile testing with the Bose Endura TEC ELF 3200 system (Bose, USA). Dry samples (*n* = 5) were used to test the mechanical properties of pure PGS with different pores, while wet samples (*n* = 5) were used when comparing the mechanical properties of cACM, gMVs‐cACM grafts, and pure PGS (wet). All the samples were trimmed into the uniform size of 0.5 × 2 × 10 mm^3^ before being gripped with custom titanium clamps. Uniaxial tensile force was applied to each segment at a rate of 2 mm min^−1^ with initial force of 0.05 N until rupture, and peak stress was defined as UTS. The biomechanical analyzer recorded the load and elongation of the sample at real time. Suture retention strength was measured on samples (*n* = 5) by placing a 5‐0 Prolene suture ≈1 mm from the end of the scaffold. The suture was fixed into the upper hook with the scaffold immobilized in the lower hook. The force needed to pull the suture apart from the scaffold was measured using a speed of 2 mm min^−1^. Maximum tensile forces were recorded for statistical analysis.

### Cell Isolation and Culture

All experimental protocols involving human cells and animal experiments were approved by the Ethics Committee of the Fourth Military Medical University. In different experimental sections throughout the study, cells were harvested from different donors, while the same batch of cells was used for producing grafts in one experiment, so that the results would be comparable and consistence in biological properties.

Chondrocytes (CHs) were isolated from auricular cartilage of 4‐weeks old New Zealand rabbits, as per the procedure described previously.^[^ ^35^ ^]^ In brief, after the auricular cartilage was dissected and sterilized, it was minced into small fragments, digested in 0.2% collagenase II in 37 °C for 12 h. The digested cell suspension was filtered and centrifugated at 1000 rpm for 5 min. Primary chondrocyte pellet was washed 2 times with PBS and resuspended in Dulbecco's modified Eagle medium (DMEM) high glucose supplemented with 15% fetal bovine serum (FBS), L‐glutamine (292 µg mL^−1^), ascorbate‐2‐phosphate (50 µg mL^−1^), penicillin (50 µg mL^−1^), and streptomycin (30 µg mL^−1^).

Human normal gingiva samples were acquired from clinical healthy gingiva of subjects without history of periodontal disease. Hu‐GMSCs were isolated according the methods applied previously.^[^ ^46^ ^]^ In summary, the epithelial layer of the sample was removed after incubated in 0.2% dispase solution 4 ° overnight. Then the sample was minced and digested in 4 mg mL^−1^ collagenase IV at 37 ° for 2 h. The dissociated cell suspension was filtered and plated on 10 cm dishes with *α*‐minimum essential medium (*α*‐MEM) containing 10% FBS, L‐glutamine (292 µg mL^−1^), 100 mm nonessential amino acid, 550 µM 2‐mercaptoethanol (2‐ME), penicillin (50 µg mL^−1^), and streptomycin (30 µg mL^−1^). The adherent confluent cells were passaged and were used in the following experiments. ≈5 × 10^5^ cells were incubated with specific PE‐ or FITC‐conjugated mouse mAbs for human CD44, CD29, CD90, and CD34, then subjected to flow cytometric analysis for cell identified. All the primary antibodies used in the study were showed in Table S1, Supporting Information. The tripotential differentiation of the GMSCs was performed as previously described.^[^ ^53^ ^]^ For osteogenic differentiation, cells were plated in 6‐well plate for 24 h and then switched to differentiation medium (*α*‐MEM, 10% FBS, dexamethasone [0.1 mm], *β*‐glycerophosphate [10 mm], and ascorbate‐2‐phosphate [50 µg mL^–1^]). For adipogenic differentiation, cells were incubated in a 6‐well plate for 24 h, and then switched to adipogenic differentiation medium (*α*‐MEM, 10% FBS, dexamethasone [0.1 mm], insulin [10 mg mL^–1^], indomethacin [0.02 mg mL^–1^]). For chondrogenic differentiation, cell microspheres obtained by centrifugation were suspended in differentiation medium (*α*‐MEM, 10% FBS, dexamethasone [0.1 mm], insulin [5mg mL^–1^], transforming growth factor‐*β*1 [10 ng mL^–1^], ascorbate‐2‐phosphate [50 µg mL^–1^]). During the cell inducing process, the medium was changed every 3 days, and the differentiation was evaluated after 21 days by Alizarin red staining, Oil red staining, and Alcian blue staining. The images were captured with an inverted microscope.

PGS/PP scaffolds were sterilized by immersing into the 75% ethanol for 3 h, then rinsed in sterile PBS for 10 min repeating 3 times. About 1 × 10^7^ chondrocytes (the 1st passage) were seeded onto each scaffold cultured dynamically in the bioreactor for 2 weeks to form the CHs sheet‐PGS/PP (cECM) graft. The seeding ratio of CHs and GMSCs cocultured on scaffold was 4:1, under the same experimental procedure as the cECM graft.

### Decellularization

The decellularization procedure referred to the previous study,^[^ ^33^ ^]^ with some modifications. The cECM grafts were frozen at −80 °C for 3 h then thawed in sterile deionized water at room temperature. After 3 cycles of frozen‐thawed processes, the grafts were incubated in 1% SDS (PH = 8.25 ± 0.12, *n* = 3) on a shaker (60 rpm) at 4 °C, with the duration ranging from 3 to 12 h. The acquired grafts were gently rinsed in PBS and incubated with 1% Triton X‐100 for 1 h. Following repeatedly rinsed in PBS the acquired grafts were incubated in DMEM containing dissolved deoxyribonuclease enzyme (50 U mL^−1^, Invitrogen) for 1 h. The cACM‐PGS grafts were ready for mechanical properties, histological analysis, examination by SEM, TEM, cell experiments in vitro and animal surgery in vivo. The whole decellularization process was sterile to ensure the operation for subsequent cell experiments and in vivo transplantation.

### Quantitative Assay of Composition of the Extracellular Matrix‐ and Acellular Extracellular Matrix‐ Grafts (DNA, Glycosaminoglycan, and Collagen)

To examine the adhesion and proliferation of CHs and GMSCs on the grafts, as well as, the effect of decellularization, nucleic acid concentration was measured with the DNeasy assay kit (Qiagen, USA). In brief, samples with the same base area were digested with proteinase K followed by a series of rinsing and purification steps according to the manufacturer's instructions, and DNA was quantified by taking absorbance readings at 260 nm using a spectrophotometer (Epoch, Bio‐Tek, USA). The tests were performed in quintuplicate, and values were normalized to the original weight (ng mg^−1^).

Sulfated GAG in grafts were quantified using Blyscan sGAG assay kit (B1000; Biocolor, UK). In brief, samples were minced into small pieces and digested with the papain extraction reagent (Sigma) at 65 °C for 18 h, then sGAG was sufficiently extracted in the supernatant. sGAG concentration (µg mg^−1^) was further measured according to the kit protocol and calculated from the sGAG standard curve.

Similarly, collagen content was quantified using Sircol Collagen assay kit (S1000; Biocolor). After minced into small pieces (1 mm^2^ size), the samples were digested with pepsin (1 mg mL^−1^ in 0.5 mm acetic acid, MP Biomedicals, Santa Ana, USA), then collagen was dissolved in the supernatant, the collagen concentration was measured according to the kit instructions. Total collagen per sample weight (µg mg^−1^) was calculated from the collagen standard curve.

### Scanning Electronic Microscopy and Transmission Electron Microscopy Examination

The preparation of samples which were processed to be examined by SEM (S‐4800, Hitachi, Japan) and TEM (JEM‐1230, JEOL, Japan) followed the standard procedures of the State Key Laboratory of Military Stomatology. For SEM examination, the samples were fixed with 3% (v v^–1^) glutaraldehyde overnight, then dehydrated with ethanol (from 30% to 100%) and dried in the hexamethyl disilazane. Samples were sputter coated with gold and observed by SEM. For TEM examination, the samples were fixed with 2.5% (v v^–1^) glutaraldehyde overnight, and observed by TEM following embedding, sectioning, and staining procedures.^[^ ^54^ ^]^

### Silver Staining and Western Blot

Samples of ECM‐ and ACM‐ grafts were minced into fragments, then RIPA cell lysis buffer including a protease inhibitor (PMSF) and a phosphatase inhibitor cocktail (Sigma) was added. The ultrasonic lysis apparatus was used to accelerate dissolving of protein from the fragments, following by protein quantification using BCA assay kit. The concentration of protein solution was adjusted to the same with RIPA solution and *β*‐mercaptoethanol. Silver staining of gels was performed using the Silver Stain Kit according to the manufacturer's instruction and visualized using Chemi DOC XRS+ (Bio‐Rad, USA). In order to identify the protein composition of different ECM, pure GMSCs cell sheet (gECM) was also included. For identifying the three marker proteins of EVs, 30 µL of protein lysate were used and separated by 10% SDS‐PAGE gels (1.5 mm thick). SDS‐PAGE gels (1.0 mm) and 20 µL protein solution were used for other Western blot tests in the study. Western blot was carried out as previously published.^[^ ^55^ ^]^ PVDF membranes were incubated with the primary antibodies overnight at 4 °C then in the horseradish peroxidase conjugated secondary antibodies and visualized on film using enhanced chemiluminescence as per the manufacturer's instructions. Band intensity was quantified using Image J software. All experiments were repeated at least 3 times.

### Isolation of gMVs and Nano‐Particle Tracking Analysis

The gMVs were isolated according to the protocol as reported previously.^[^ ^56^ ^]^ Briefly, the gMVs‐cACM sample (1.5 × 1.5 cm^2^, *n* = 6 independent samples) was minced into small pieces of ≈1.0 × 1.0 mm^2^ on ice. Following incubation in mixed medium containing the collagenase type II (0.1%) under mild agitation at 37 °C for 30 min, the tissue solution was fully filtrated with a 70‐µm cell strainer and subjected to successive centrifugations at 300 g × 10 min, 2000 g × 20 min, and 16 500 g × 20 min to remove collagen fibril remnants. The supernatant was then centrifuged at 100 000 g for 2.5 h at 4 °C. After removing the supernatant carefully and re‐suspend the pellet (gMVs) in 500 µL PBS, analysis of absolute size distribution of gMVs was performed using Nano Sight NS 300 (Malvern Ltd., UK).

### Evaluating In Vivo Toxicity of the gMVs‐cACM Grafts

Two gMVs‐cACM patches (0.5 × 1 cm^2^) were implanted subcutaneously on the back of the BALB/C mouse (implanting in the symmetrical position on the left and right, *n* = 5 in each group). The tissue around the grafts were harvested at 1 and 3 weeks postoperatively. Moreover, the heart, liver, spleen, lung, kidney, and brain were also harvested for evaluating systematic toxicity at 1 and 3 weeks. All the harvested tissues were embedded in paraffin, sectioned, and evaluated by H&E staining.

### The Examination of the gMVs Release from Grafts In Vitro and In Vivo

The PGS, cACM, and gMVs‐cACM graft were stained by DiO (5 µM) according to the protocol. For in vitro test, the grafts were placed in the upper chamber of the transwell plate, while the HBEs were seeded on the cell climbing pieces placed in the lower chamber. The cell climbing pieces were fixed and stained by DAPI at 1 d, 3 d, and 7 d after coculturing, then observed under laser confocal microscope. The number of the DiO labeled gMVs endocytosed by HBEs was calculated from two random high‐magnification fields from each of three independent samples. The in vivo release of gMVs from gMVs‐cACM graft was monitored in the subcutaneous tissue of mice ventral neck, through bioluminescence imaging recorded at the 1, 3, 7 days after implantation (*n* = 3 in each group), at 490 nm excitation wavelength by in vivo imaging System (IVIS Lumina XRMS, Series III).

### Proteomics Analysis

Label free LC‐MS/MS was run to identify the proteomics of the cACM graft and the gMVs‐cACM graft (*n* = 3 in each group). Briefly, after protein extraction and trypsin digestion, the peptides were dissolved in liquid chromatography mobile phase A and separated by NanoElute ultra performance liquid chromatography system, then injected into Capillary ion source for ionization analyzed by tims‐TOF Pro mass spectrometry. The resulting MS/MS data were processed using Maxquant search engine (v.1.5.2.8). KEGG online service tools KAAS was used to annotate the differential protein, then matched into corresponding pathways in the database through the KEGG Mapper. GO annotation proteome was derived from the UniProt‐GOA database (http://www.ebi.ac.Uk/GOA/). For further hierarchical clustering based on differentially expressed protein functional classification, it was visualized by a heat map. A two‐tailed Fisher's exact test was employed to test the enrichment of the differentially expressed protein against all identified proteins, with a corrected *p*‐value < 0.05 is considered significant. The proteome sequencing analysis of tissue samples from in vivo experiment was the same as the above.

### Enzyme‐Linked Immunosorbent Assay

The proteins extracted from the two ACM grafts and the CM of the cultured ECM graft were collected to examined with ELISA kits of the expression levels of VEGF, KGF, and TGF‐*β*1 following the manufacturer's guidance. The absorbance value at 450 nm was measured using a microplate reader and the concentration was calculated according to the standard curve.

### Effects of Grafts on Migration and Proliferation of Human Bronchial Basal Epithelial Cellss In Vitro

HBEs was donated by the Department of Thoracic surgery of the Second Affiliated Hospital of the Air‐Force medical university. EV free medium was used in this section. The cell viability of HBEs seeded directly on the graft was evaluated with the CCK‐8 assay. About 2.5 ×10^4^ HBEs were seeded on each graft mentioned above which were set in the 24‐well plates. The CCK‐8 solution was added in on day 1, 3, 5, 7 of the culture durations. After incubation for 1 h, the absorbance value at 450 nm was read with a spectrophotometer. After the last CCK‐8 test, DNA content of HBEs cultured on the graft was quantified according to the protocol above.

A scratch wound healing migration assay was performed to verify the effects of ACM graft on HBEs. Grafts were grouped to cACM graft, gMVs‐cACM graft, and gMVs^(‐)^‐cACM graft. The gMVs^(‐)^‐cACM graft is a modified gMVs‐cACM graft, during the coculture of GMSCs and chondrocytes on PGS, the production of GMSCs derived gMVs was inhibited by administration of GW4869 (0.017 mm), so that the ECM lacking gMVs was acquired, and decellularized construct was named as gMVs^(‐)^‐cACM graft. The gMVs ^(‐)^‐cACM group was set to detect whether the bioactivity of the gMVs‐cACM graft was affected after inhibiting the release of EVs by GW4869. Grafts were placed on upper chamber with 4 µm pore size of 24‐well Transwell plate, while HBEs were seeding in the lower chamber with DMEM low glucose media containing 2.5% FBS which was EVs free. To test participation of the JAK2‐STAT1 signal pathway and TGF‐*β*1 in the promoting effects on HBEs in different in vitro experiment, AG490 (10 mm) and the neutralizing antibody of TGF‐*β*1 (500 ng mL^–1^) was added in the culture media of lower chamber in the gMVs‐cACM group, respectively. A scratch was created to simulate a wound in a cell monolayer. The changes of the wound area were compared among groups over time on the 1st day and 2nd day using a motorized inverted microscope (IX71, Olympus, Japan). The migration rate was calculated by Image J as per the previous method.^[^ ^57^ ^]^

To test the biological effects of GMSC‐derived EVs (gEVs) on HBEs in vitro, gEVs were isolated according to the previous studies,^[^ ^58^ ^]^ and the protein concentration of gEVs was determined by BCA kit. HBEs were treated with medium (2.5% FBS), media containing gEVs (10 µg mL^–1^, 20 µg mL^–1^), respectively.

The proliferation of HBEs was determined by the Ki67 immunofluorescence staining at 48 h after coculture. HBEs were seeded on the cell climbing pieces placed in the lower chamber, with ACM grafts placed in the upper chamber of 24‐well Transwell plate. Western blotting was performed to detect the markers of cell migration and cell proliferation, including CD44, MMP9, Cyclin D1, and Axin2.

### Animal Grouping and Surgery

Animal experiment was approved by the Institutional Animal Care and Use Committee of the Fourth Military Medical University, Xi'an, PR China. The operative procedure and animal care were performed in accordance with the institutional guidelines of the committee. In brief, total 48 New Zealand rabbits (3–4 months old, 2.8–3.5 kg) assigned randomly into PGS graft (*n* = 6), cACM graft (*n* = 21), and gMVs‐cACM graft (*n* = 21). The animals were anesthetized with intramuscular injection of xylazine hydrochloride (0.05 mg kg^−1^) and 3% pentobarbital sodium (15 mg kg^–1^) (Sigma, USA). The partial anterolateral tracheal defect model was prepared on the normal trachea by cutting estimated area of tracheal wall with size of 5 × 10 mm^2^, and graft was implanted with 5‐0 poly‐glyconate suture onto the defect. After the operation, penicillin (2 × 10^4^ U kg^−1^) was injected daily last for 3 days.

### Gross Morphology and Micro‐CT Scan

Animals of each group were sacrificed by injecting overdosage of pentobarbital sodium, then the reconstructed tracheas were harvested and evaluated grossly. Tissue samples were photographed, then micro‐CT (Inveon, Siemens, Germany) examination was performed. The patency of the trachea was shown by the reconstructed images of sagittal plane, coronal plane, and transverse plane. To quantitatively evaluate the degree of trachea stenosis, 3D reconstruction image was used to calculate the ratio of stenosis site volume to total tracheal volume at the site of the defect.

### Histological Analysis

The harvested grafts were embedded in optimum cutting temperature compound (OCT, Sakura Finetek, USA) immediately, sectioned into slice with 5–8 µm thickness and transferred onto slides. The sections were fixed with 4% paraformaldehyde for 10 min, rinsed, and then stained with H&E for general histology, Safranin‐O to analyze GAG distribution and Masson trichrome (MTS) to examine the expression of collagen fiber.

In the animal experiment, harvested samples were fixed in 4% formaldehyde for 24 h, following with dehydration in graded alcohols. Then samples were embedded in paraffin and sectioned into slices of 5 µm thickness. Sections were then stained with H&E for general histology, Pentachrome staining with modified for marking goblet cells and ciliated columnar cells.

### Immunofluorescence Staining

Immunofluorescence staining was performed as previously described. The grafts seeded with HBEs were fixed in cold acetone for 20 min, washed with PBS, and permeabilized in 0.5% Triton‐X solution for 20 min at room temperature (RT). Then grafts were incubated with 10% goat serum (Boster, Wuhan, China) for 30 min at RT, following with primary antibodies (CK5 + 8, Abcam, USA) overnight at 4 °C. After rinsed with PBS, stained with secondary antibodies for 60 min at 37 °C, and then mounted with DAPI. Ki67 was stained for labeling proliferated HBEs on the cell crawling pieces.

Histological immunofluorescence staining was also used to examine the expression of CK5+8, Mucin‐5AC, and *β*‐Tubulin‐IV for epithelial basal cells, goblet cells, and ciliated columnar cells in the regenerated tracheal tissue respectively. Endothelial cell was observed using anti‐CD31 staining to verify the vascularization of neo‐mucous layer. To manifest the participation of JAK2‐STAT1 pathway in the repair of tracheal defect, STAT1 and p‐STAT1 antibody were stained.

The positive cells were visualized using a confocal laser scanning microscope (FV 1000, Olympus, Japan) and measured with Image J software. The quantification of positive cell number was collected from 6 random high magnification vision fields (200X) of three independent samples in each group. The rate of epithelialization was calculated by the Image J as a ratio of the length of CK5 + 8 positive cell line to the length of the defect at cross section in six low magnification vision fields (40X) from three independent samples.

### Statistical Analysis

Statistical analyses were performed using SPSS software (version 18.0) and Graphpad Prism 8.0 software. The continuous variables were expressed as mean ± standard deviation (SD). For comparison between two groups, the means were compared using Student's *t*‐test. The difference between multiple groups was determined by one‐way analysis of variance (ANOVA) followed by Tukey's post hoc analysis. *p* < 0.05 was considered statistically significant.

## Conflict of Interest

The authors declare no conflict of interest.

## Supporting information

Supporting Information

## Data Availability

The data that support the findings of this study are available from the corresponding author upon reasonable request.
