# Supplementary material for: Cartilaginous Extracellular Matrix Enriched with Human Gingival Mesenchymal Stem Cells Derived “Matrix Bound Extracellular Vesicles” Enabled Functional Reconstruction of Tracheal Defect
Source: Adv Sci (Weinh). 2021 Nov 28;9(2):2102735. doi: 10.1002/advs.202102735 (PMC8805569; doi:10.1002/advs.202102735)
Supplement: Supplementary file 1 — Supporting Information [file ADVS-9-2102735-s001.pdf]

ADVANCED SCIENCE
Open Access

Supporting Information

for *Adv. Sci.,* DOI: 10.1002/advs.202102735

Cartilaginous ECM enriched with Hu-GMSCs derived "matrix bound EVs" enabled functional reconstruction of tracheal defect

Tian Zeng, Pingping Yuan, Lirong Liang, Xinchi Zhang, Hui Zhang*, Wei Wu*

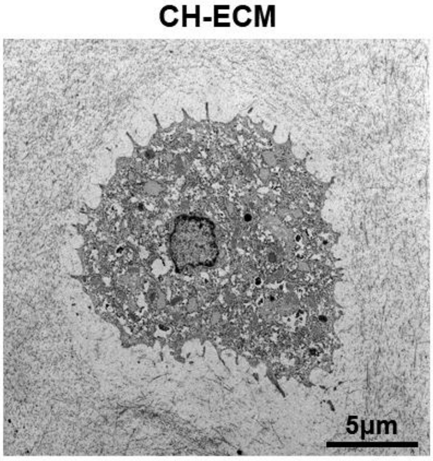

Figure S1. TEM examination showed that the ECM formed by chondrocytes was mainly composed of fibrous collagen.

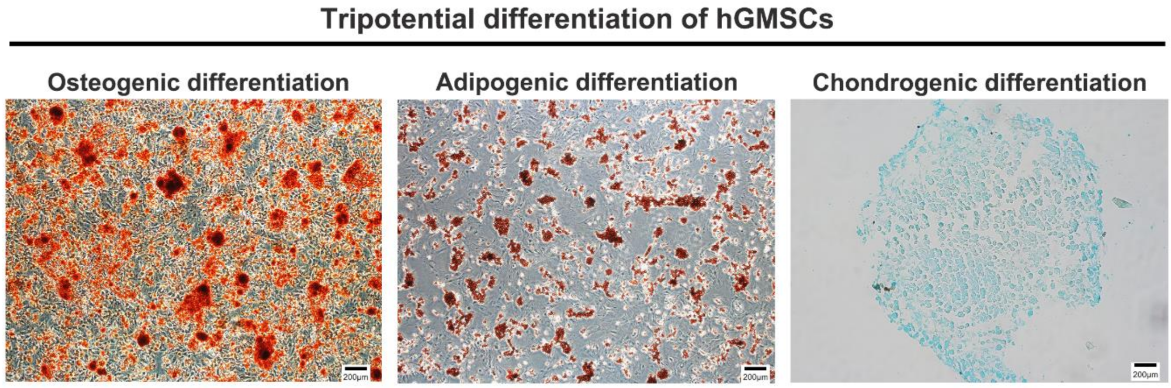

Figure S2. Tripotential assessment of GMSCs. Images illustrating GMSCs tripotential differentiation were identified with Alizarin red (osteocytes), Oil Red O (adipocytes) and Alcian Blue (chondrocytes).

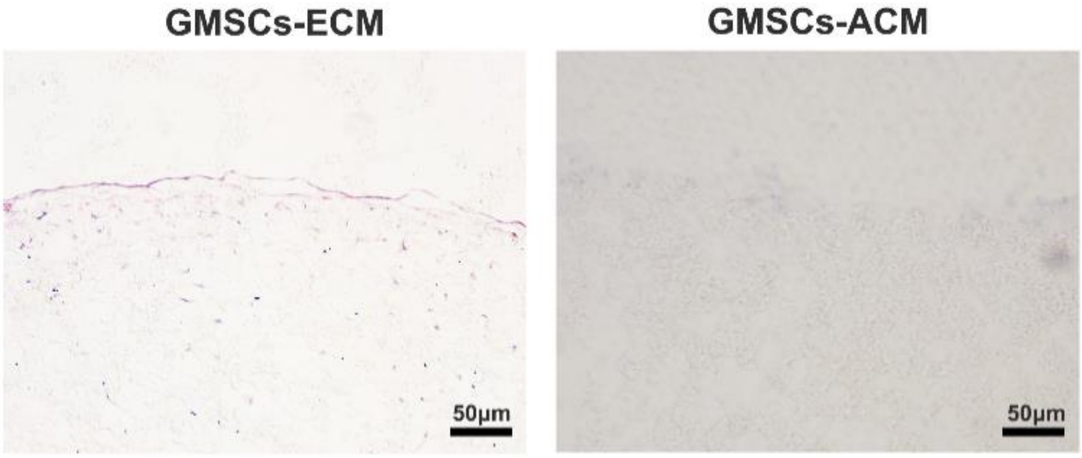

Figure S3. H&E staining of the GMSCs derived ECM before and after decellularization. GMSC can only form small amount of ECM, which can hardly anchor porous PGS after decellularization.

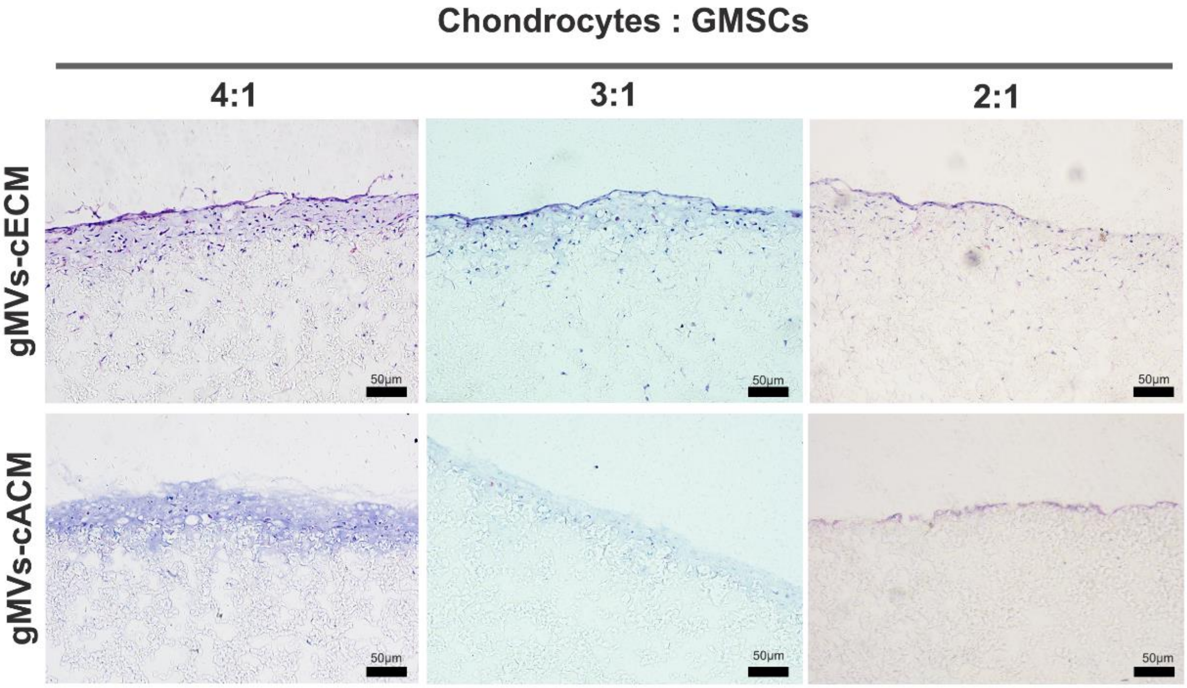

Figure S4. H&E staining of gMVs-cECM and gMVs-cACM constructs produced with different chondrocytes/GMSCs ratios, which showed compromised ECM integrity and quality in 2:1 and 3:1 group, especially after decellularization. 4:1 ratio acquired sufficient cartilaginous ECM, which well anchored on porous PGS after decellularization

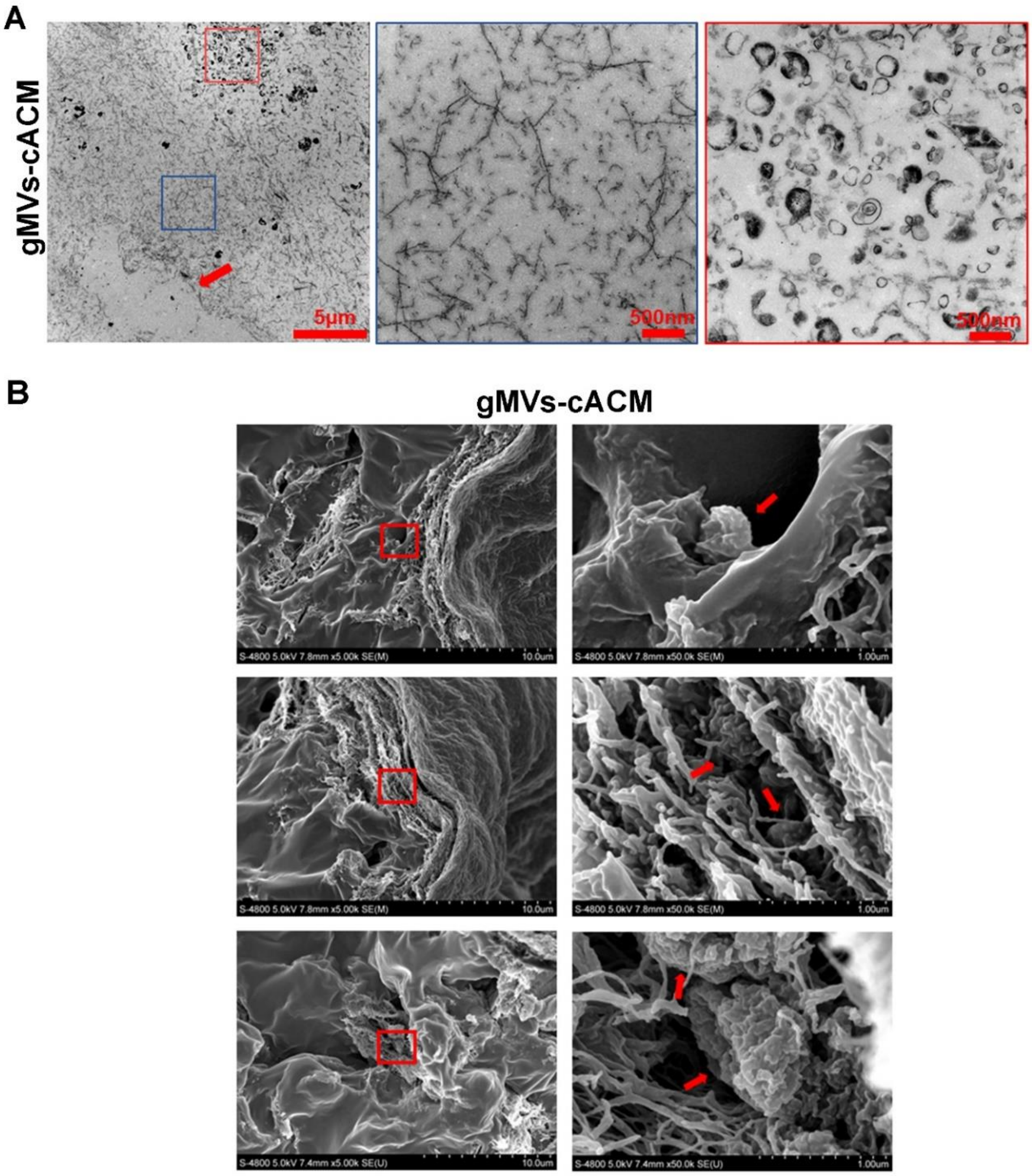

Figure S5. TEM and SEM examination showed the distribution of gMVs in cACM graft. (A) TEM examination showed the gMVs were mainly distributed at a slightly distant location from the lacuna of cell (Red arrow). (B) SEM examination showed the gMVs (Red arrows) were widely distributed on the gMVs-cACM graft, such as in the pores of the PGS and in the fibers of the gMVs-cACM.

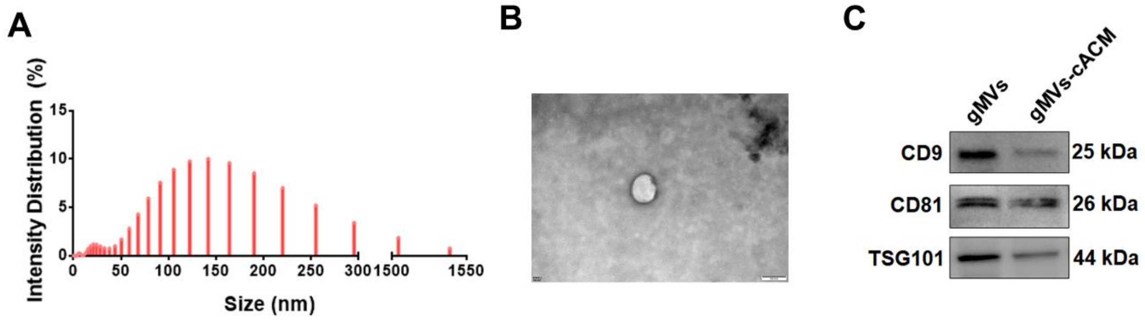

Figure S6. Identification of gMVs extracted from gMVs-cACM. (A) The gMVs size and intensity distribution were tested by NTA. (B) TEM images showed typical bilayer-membrane structure of gMVs. (C) Western blot was performed for identifying three exosomal surface markers: CD9, CD81 and TSG101.

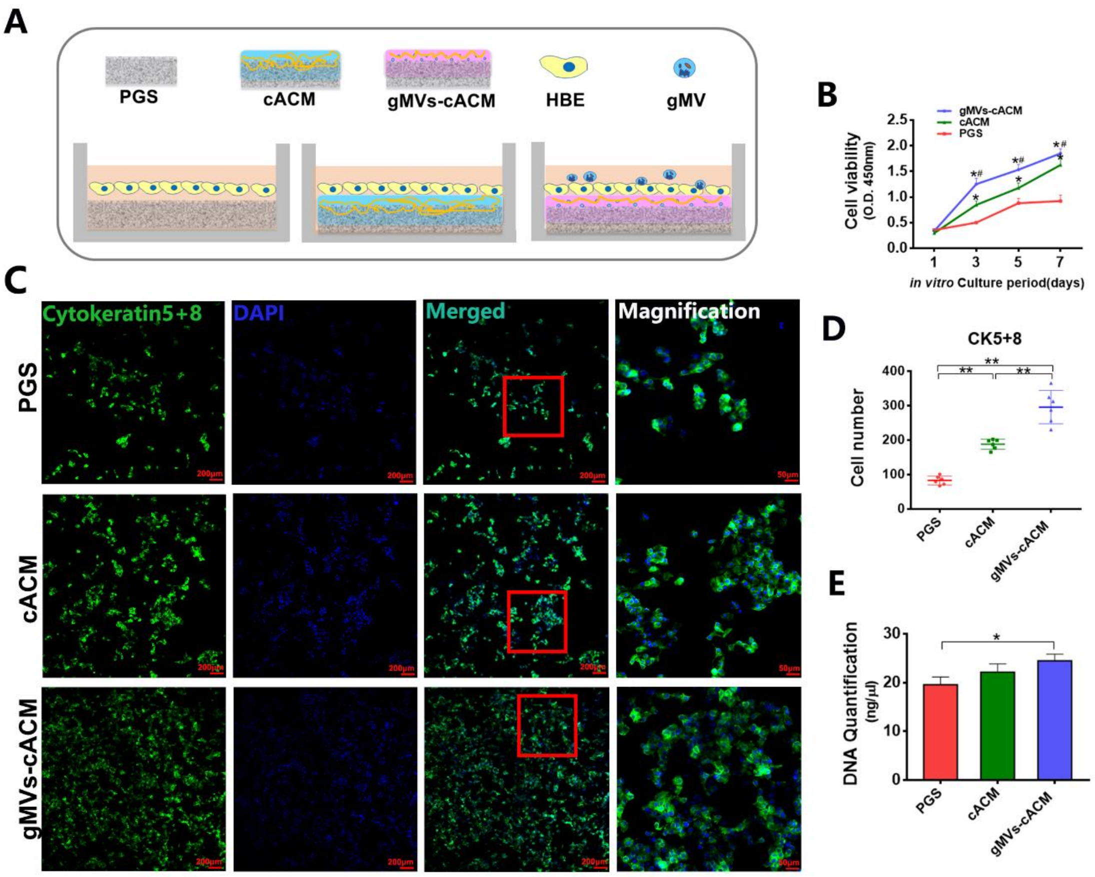

Figure S7. The gMVs-cACM graft promoted the proliferation of HBEs *in vitro*. (A) Schematic illustration of HBEs seeding on the PGS, cACM and gMVs-cACM grafts. (B) CCK-8 assay revealed that the cell viability of HBEs seeded on the gMVs-cACM graft was significantly higher than that on the other two grafts from the 3rd day to 7th day (n = 6 independent samples, *: *vs* PGS group, *: P < 0.05; #: *vs* cACM group, #: P < 0.05). (C) Immunofluorescent staining of HBEs (CK5+8: green; DAPI: blue) on PGS, cACM and gMVs-cACM grafts at 7 d after co-culture. (D) The number of HBEs on the gMVs-cACM graft was highest among the three groups (n = 6 independent samples, **: P < 0.01). (E) DNA quantification revealed HBEs on the gMVs-cACM graft proliferated more than that on the other two grafts (n = 3 independent samples, *: P < 0.05). Data were represented as the mean ± SD for each group and significance was determined by one-way ANOVA followed by Tukey's post hoc analysis.

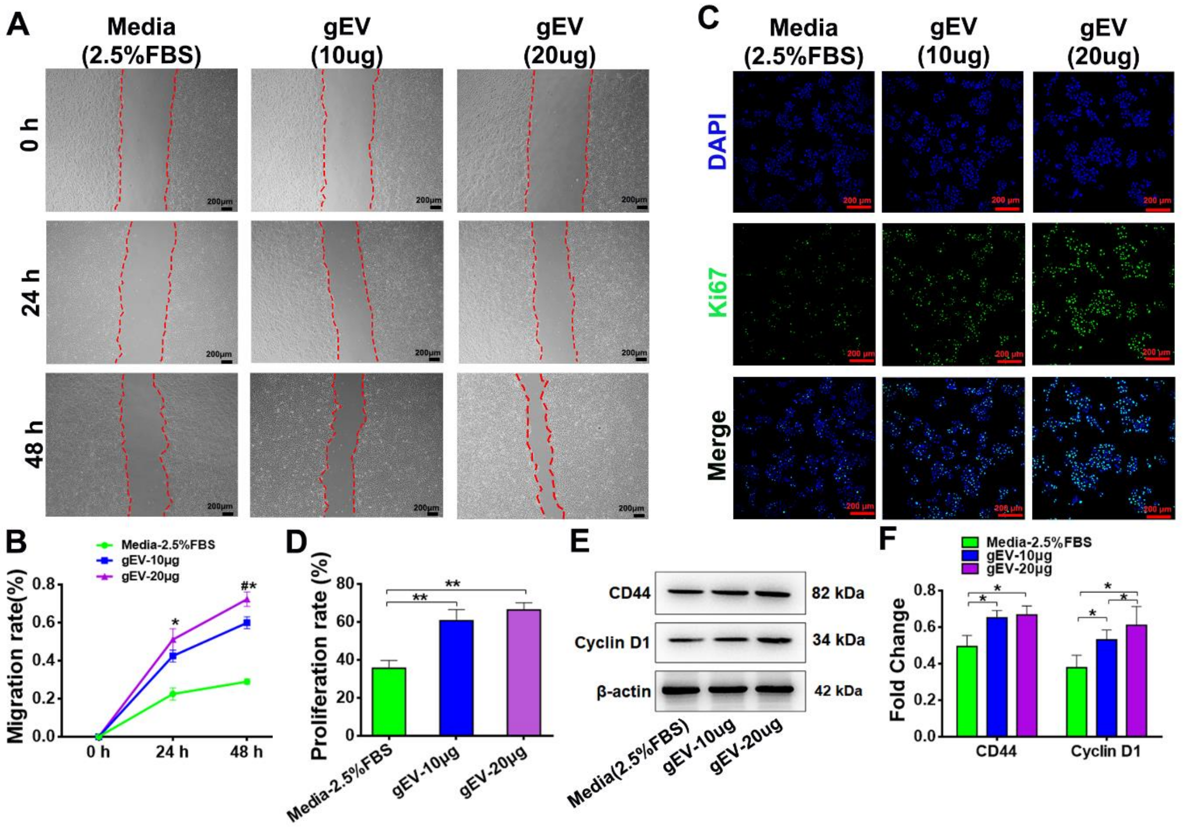

Figure S8. GMSC-derived EVs showed the promoting effects on the migration and proliferation of HBEs. (A, B) The gEVs treatment enhanced migration of HBEs in a dose-dependent manner in the cell scratch assay, quantitatively analysis of the migrating rates of HBEs in different groups at the 24th h and 48th h was performed (n = 6 independent samples, *: vs media-2.5%FBS group, *: $P < 0.05$; #: vs gEVs-10μg group, #: $P < 0.05$). (C) Ki67 immunofluorescence staining revealed the increased proliferation of HBEs in gEVs group at 48 h. (D)The proliferation rate of HBEs in gEVs-20μg group was the highest among the three groups based on number of Ki67+ cells after 48 h of co-culture (n = 6 independent samples, **: $P < 0.01$). (E, F) Western blotting showed the high expression of CD44 and Cyclin D1 in HBEs treated with the gEVs (n = 3 independent samples, *: $P < 0.05$). Data were represented as the means ± SD for each group and significance was determined by one-way ANOVA followed by Tukey's post hoc analysis.

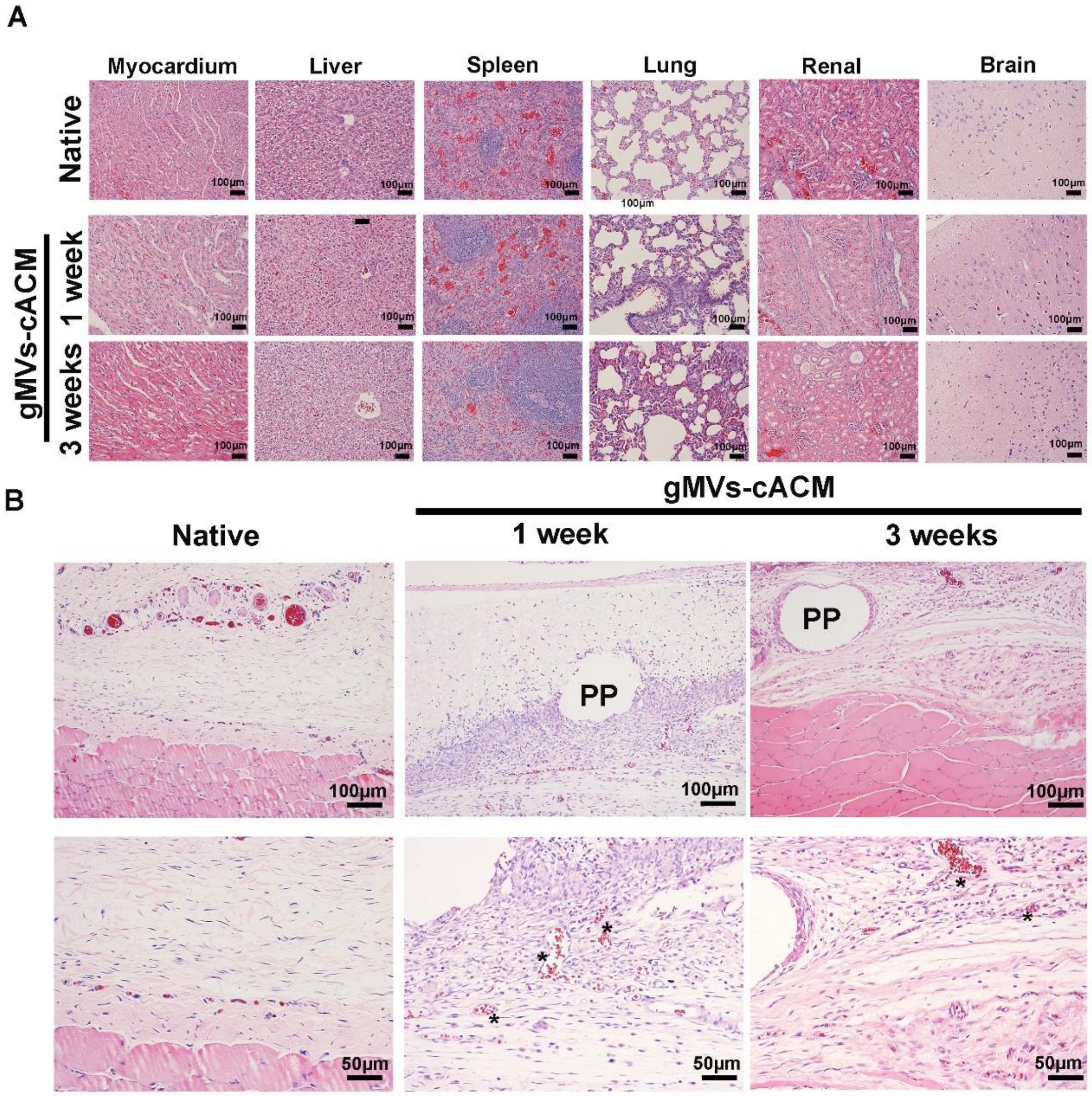

Figure S9. Evaluation of *in vivo* toxicity following gMVs-cACM grafts implantation. (A) H&E staining of important organs such as myocardium, liver, spleen, lung, renal and brain showed no pathological changes at 1 week and 3 weeks. (B) Mild inflammation was identified around grafts at 1 week while diminished at 3 weeks, accompanying with active cell ingrowth and angiogenesis in grafts. Low-magnification images presented on upper line and high-magnification images on lower line. Neo-capillaries were marked with the black stars.

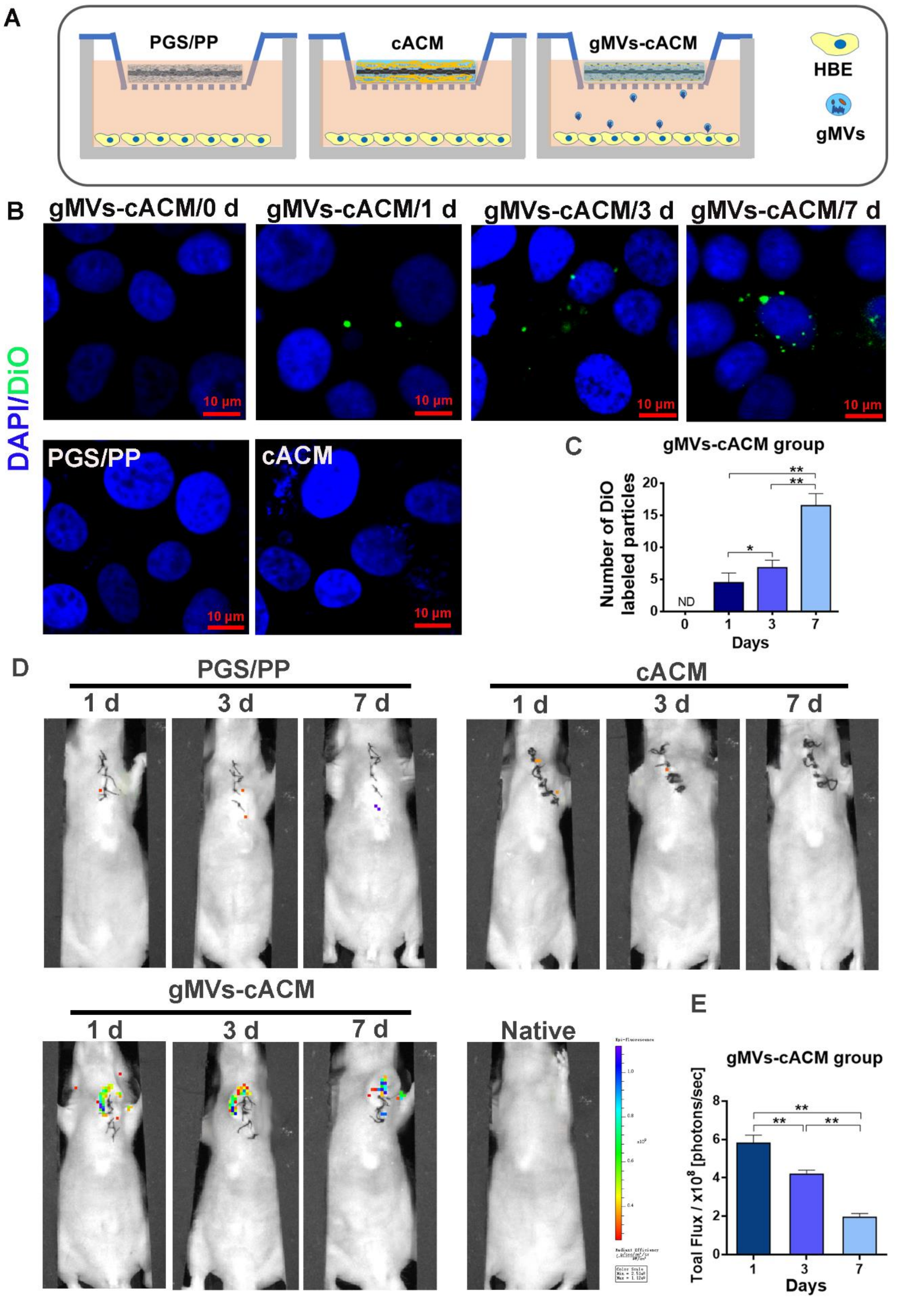

Figure S10. Monitoring the release of gMVs from grafts *in vitro* and *in vivo.* (A) Schematic illustration of the co-culturing HBEs with PGS/PP, cACM graft and gMVs-cACM graft stained by DiO. (B) The DiO labeled gMVs were released from the gMVs-cACM graft into

the media and engulfed by HBEs *in vitro.* (C) The quantification of the DiO labeled gMVs (n = 6 independent samples, *: $P < 0.05$, **: $P < 0.01$). (D) The DiO stained gMVs-cACM grafts were visualized by live animal imaging at implanting region at 1 d, 3 d and 7 d, PGS/PP and cACM groups were also examined as controls. (E) Quantitative analysis of total flux at the implantation site using IVIS imaging (n = 3 independent samples, **: $P < 0.01$). Data were represented as the means ± SD for each group and significance was determined by one-way ANOVA followed by Tukey's post hoc analysis.

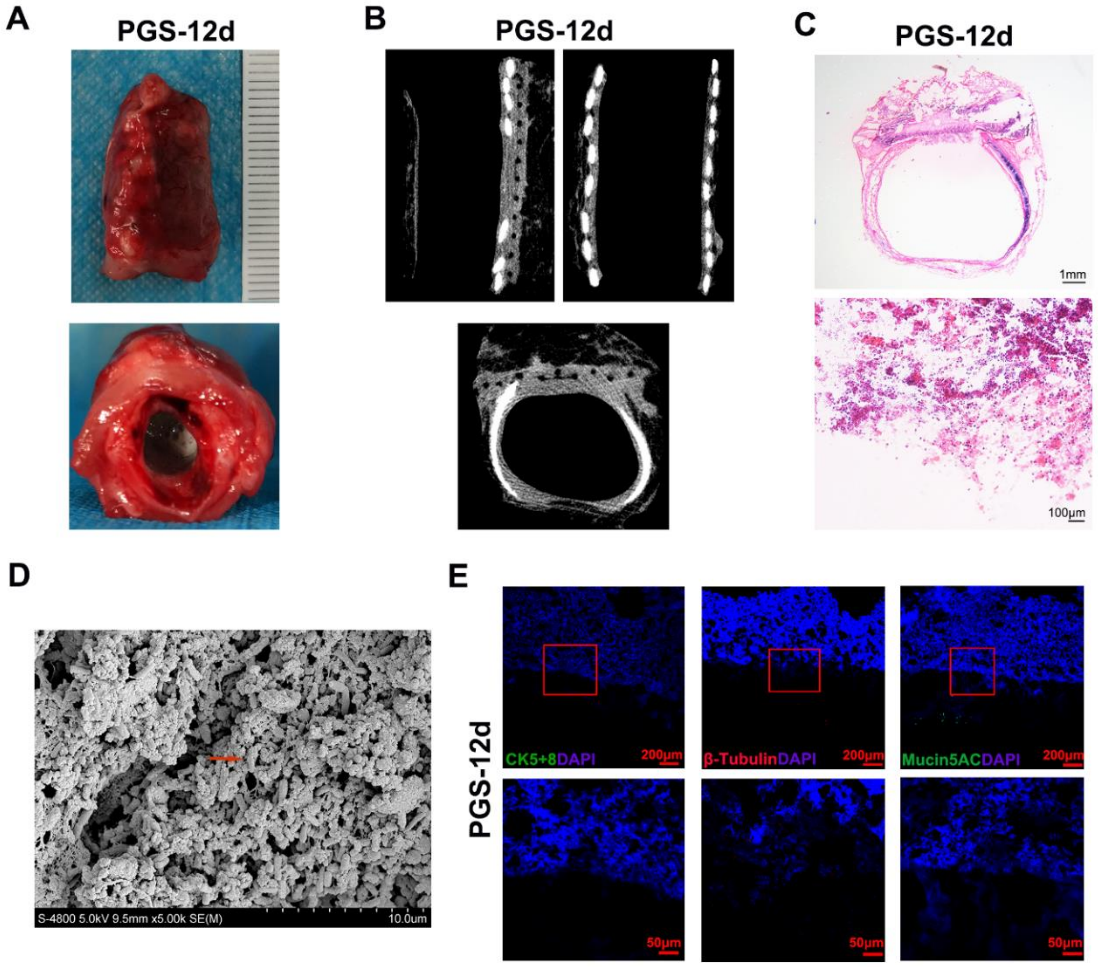

Figure S11. Data of the animal died on the 12th day after transplantation in PGS group. (A) The gross view showed the reconstructed trachea was blocked by the sputum. (B) CT images showed the luminal morphology and tracheal wall of the regenerated trachea. (C) H&E staining of the reconstructed trachea showed the infiltration of inflammatory cells in the luminal side of the PGS graft without neo-epithelium. (D) SEM examination showed a large number of bacteria (Red arrow) attached on the luminal side of the PGS graft. (E) Immunofluorescence staining of CK5+8, β-Tubulin-Ⅳ and Mucin5AC verified no neo-epithelium in the defect site of the PGS group.

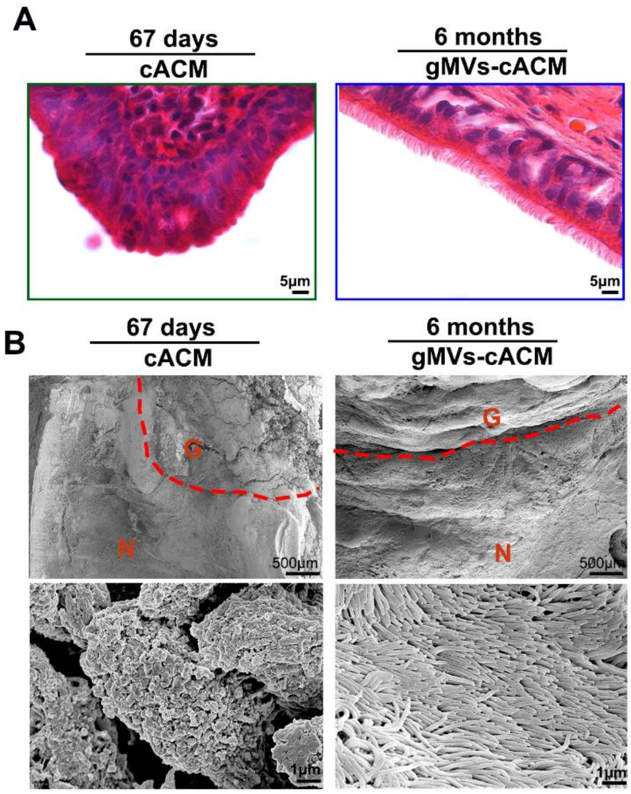

Figure S12. Comparison of neo-epithelium between cACM group and gMVs-cACM group at the end point of observation. (A) The morphology of the regenerated epithelium on the luminal side of cACM group and gMVs-cACM group was shown by H&E staining at high magnification. (B) SEM examination showed the microstructure of the regenerated epithelium on the luminal side of the graft in cACM group and gMVs-cACM group at the end point (Red dotted line: the junction of the graft and the autologous trachea; G: graft; N: native).

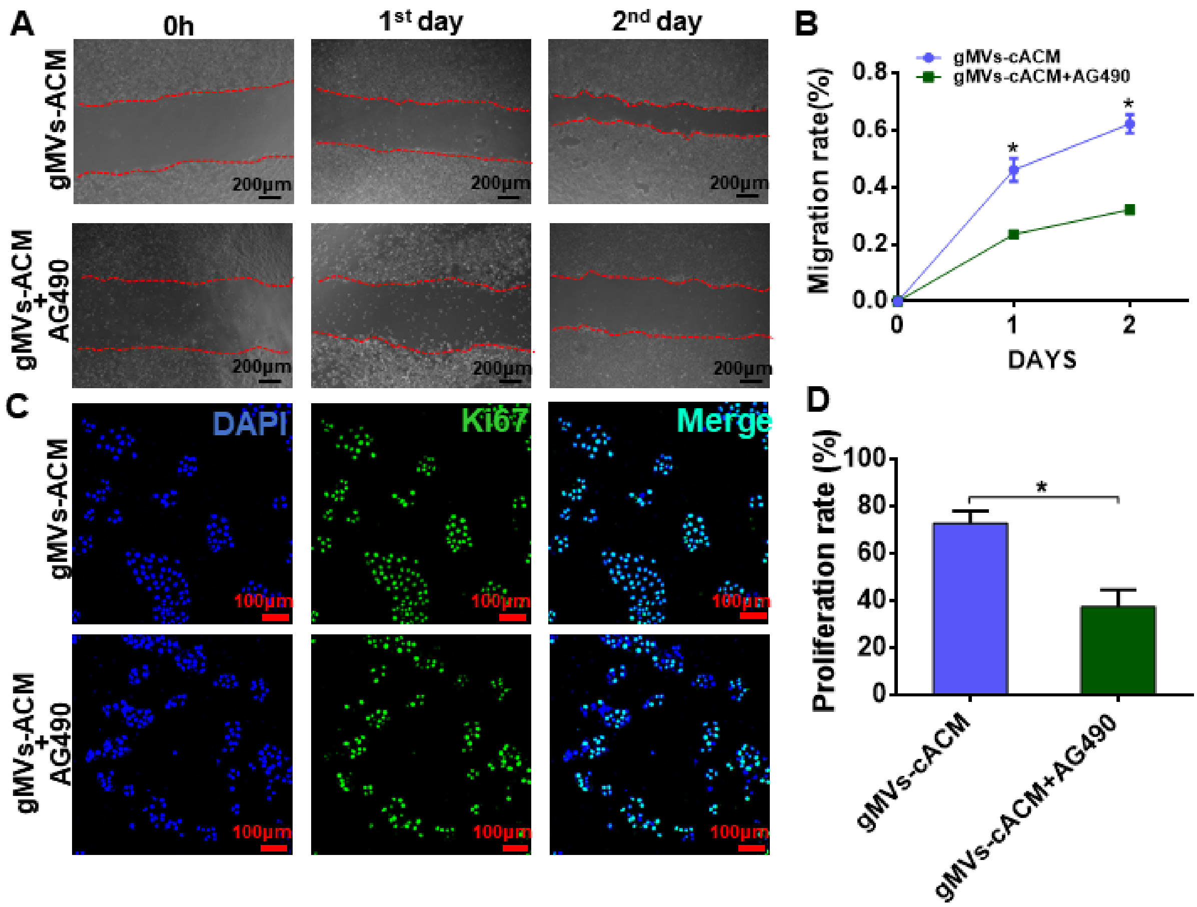

Figure S13. The participation of JAK2-STAT1 signaling pathway on HBEs migration and proliferation induced by the gMVs-cACM graft was verified *in vitro*. (A and B) The cell scratch assay suggested that the inhibition of JAK2-STAT1 signaling pathway by AG490 declined the promoting effects of gMVs-cACM graft on the migration of co-cultured HBEs from the 1st day to the 2nd day (n = 6 independent samples, *: $P < 0.05$). (C and D) Inhibition of JAK2-STAT1 signaling pathway partially counteracted the proliferation of HBEs induced by gMVs-cACM graft at 48 h (n = 6 independent samples, *: $P < 0.05$). Data were represented as the means ± SD for each group and the significance was determined by the student t-test analysis.

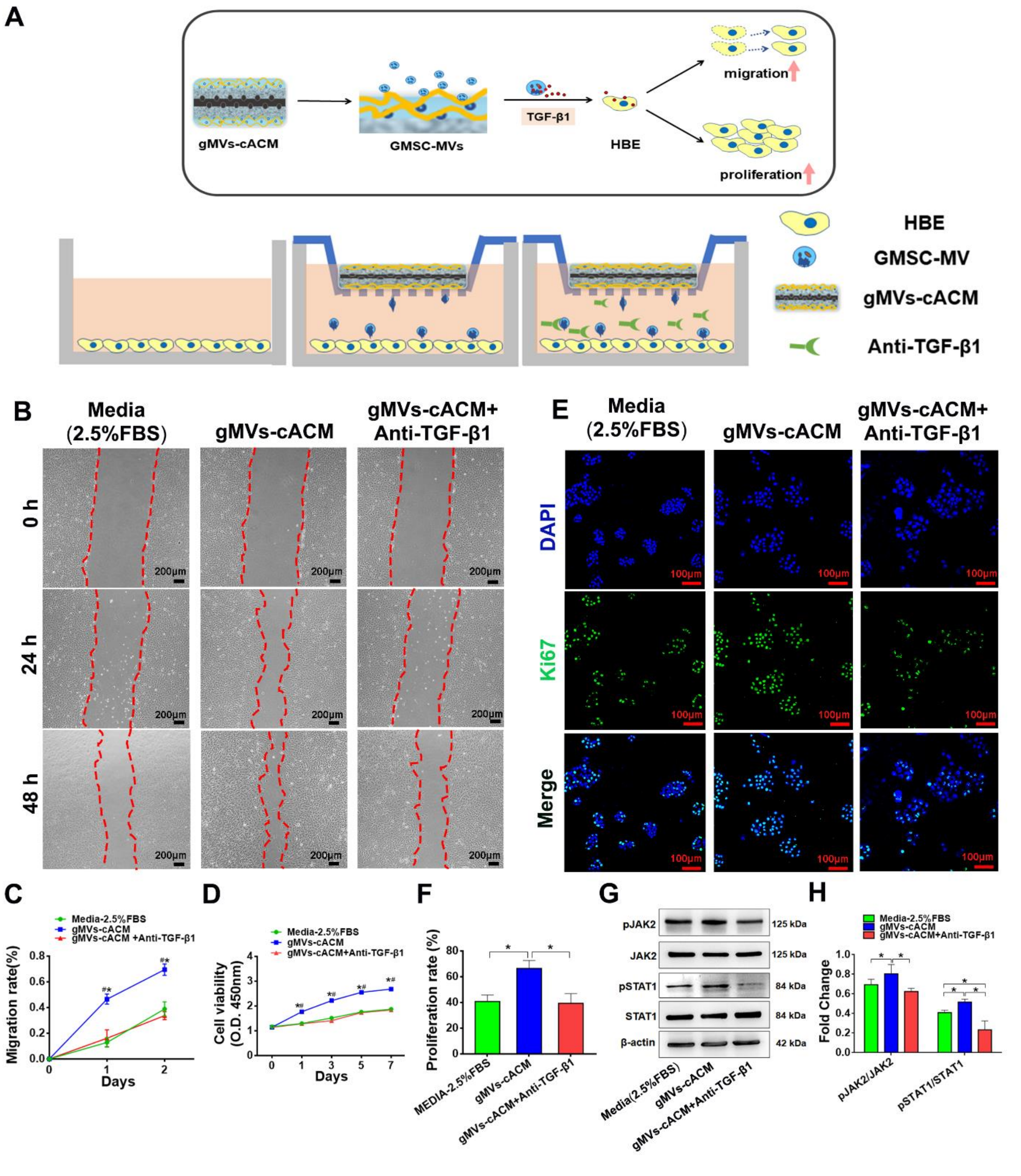

Figure S14. The gMVs-cACM grafts may promote the proliferation and migration of HBEs through TGF-β1 mediated JAK2-STAT1 signal pathway activation. (A) Schematic illustration of the HBEs cocultured with gMVs-cACM graft by Transwell plates. (B, C) The cell scratch assay revealed the promoting effect of gMVs-cACM graft on the migration of HBEs was significantly inhibited by neutralizing antibody of TGF-β1 (n = 6 independent samples, *: vs media, *: $P < 0.05$; #: vs gMVs-cACM + anti-TGF-β1, #: $P < 0.05$). (D) The cell viability of HBEs was higer than the other two groups at 1, 3, 5, 7 days after co-culturing (n = 6 independent samples, *: vs media, *: $P < 0.05$; #: vs gMVs-cACM + anti-TGF-β1, #: $P < 0.05$). (E, F) The proliferation rate of HBEs in gMVs-cACM group was highest among the

three groups based on positive cell number of Ki67 (n = 6 independent samples, *: $P < 0.05$). (G, H) JAK2-STAT1 signal pathway was up-regulated in the HBEs of gMVs-cACM group,while significantly weakened after feeding neutralizing antibody of TGF-β1 (n = 3 independent samples, *: $P < 0.05$). Data were represented as the means ± SD for each group and significance was determined by one-way ANOVA followed by Tukey's post hoc analysis.

Table S1. Primary Antibodies

Name	Supplier	Catalog no.	Dilution
CD29	1:100	Abcam	ab134179
CD44	1:100	Abcam	Ab189524
CD90	1:500	Abcam	ab225
CD34	1:50	Abcam	ab81289
CD81	1:1000	Abcam	ab109201
CD9	1:1000	Abcam	ab236630
TSG101	1:1000	Abcam	ab125011
Cytokeratin 5+8	1:500	Abcam	ab9005
CD44	1:1000	GeneTex	GTX102111
MMP9	1:1000	GeneTex	GTX100458
Ki67	1:200	Abcam	ab15580
Cyclin D1	1:1000	Gene tex	GTX108624
Axin2	1:1000	Abcam	ab109307
β-Tubulin-Ⅳ	1:500	NOVUS	NBP1-57005
Mucin5AC	1:500	NOVUS	NBP2-32632H
CD31	1:200	NOVUS	NB100-64796
JAK2	1:1000	Cell Signaling Technology	#3230
pJAK2	1:1000	Cell Signaling Technology	#4406
STAT1	1:1000	Cell Signaling Technology	#14994
pSTAT1	1:1000	Cell Signaling Technology	#7649
TGF-β1	1:1000	BIOTECHNE	AF-101-SP
VEGF	1:1000	Gene Tex	GTX102643
KGF	1:1000	Abcam	Ab131162
β-actin	1:1000	Gene Tex	GTX629630
